# Review on Synthesis and Properties of Lithium Lanthanum Titanate

**DOI:** 10.3390/ma16227088

**Published:** 2023-11-08

**Authors:** Alexandru Okos, Cristina Florentina Ciobota, Adrian Mihail Motoc, Radu-Robert Piticescu

**Affiliations:** National Research and Development Institute for Non-Ferrous and Rare Metals, 077145 Bucharest, Romania; crusti@imnr.ro (C.F.C.); amotoc@imnr.ro (A.M.M.); rpiticescu@imnr.ro (R.-R.P.)

**Keywords:** solid state electrolyte, lithium batteries, structure–properties correlation, lithium lanthanum titanates

## Abstract

The rapid development of portable electronic devices and the efforts to find alternatives to fossil fuels have triggered the rapid development of battery technology. The conventional lithium-ion batteries have reached a high degree of sophistication. However, improvements related to specific capacity, charge rate, safety and sustainability are still required. Solid state batteries try to answer these demands by replacing the organic electrolyte of the standard battery with a solid (crystalline, but also polymer and hybrid) electrolyte. One of the most promising solid electrolytes is Li_3x_La_2/3−x_TiO_3_ (LLTO). The material nevertheless presents a set of key challenges that must be resolved before it can be used for commercial applications. This review discusses the synthesis methods, the crystallographic and the ionic conduction properties of LLTO and the main limitations encountered through a number of selected studies on this material.

## 1. Introduction

As all articles related to the topic of lithium batteries begin, this article will start by highlighting the importance of the batteries. Lithium-ion batteries offer the highest energy density of any battery type at low weight, high power output and excellent cycling performance. The drawbacks of the Li-ion batteries consist mainly of the reliance on an organic electrolyte and the intercalation mechanism. The organic electrolyte is flammable, and therefore it represents a fire hazard. The intercalation mechanism requires the usage of a graphite anode, and this has the effect of limiting the specific capacity of the battery [1,2].

The energy density can be improved by replacing the graphite anode with a metallic lithium anode. The energy density of Li is 3860 mAh/g [1]. This refers only to Li metal so the total specific capacity when taking into account all the components of the battery is smaller than 3860 mAh/g; however, it remains superior to one of the conventional batteries. The employment of a metallic anode, however, causes further complications. During operation of the battery, Li dendrites are formed on the surface of the anode. In a relatively short time, these dendrites extend across the liquid electrolyte and separator and eventually reach the cathode, thus forming a short circuit [2]. The employment of a solid-state electrolyte can prevent the dendrites from growing. At the same time, replacing the liquid electrolyte removes the fire hazard intrinsic to the conventional batteries [3].

The solid electrolyte must accomplish at the same time a number of requirements. It must present high ionic conductivity (to allow easy transfer of Li ions from one electrode to the other), low electronic conductivity (to not cause self-discharge), high mechanical strength (to suppress dendrite growth) and chemical stability to both electrodes (to not cause decomposition, but to allow the battery to function at the highest voltage achievable) [4]. The surface chemistry must also be controlled. The ideal material should achieve a good interface contact to the electrodes, but, at the same time, should avoid the formation of the solid electrolyte interface layer (SEI), which passivates the electrodes and increases the contact impedance [3,4].

Many types of materials were studied as potential solid-electrolyte replacements for the current organic liquid type electrolytes. There are three large classes of materials that are considered solid-electrolyte. These classes are: polymer electrolytes, ceramic electrolytes and hybrid electrolytes. The characteristics of these materials are summarized in Table 1.

The polymer electrolytes generally present relatively low ionic conductivity and low mechanical strength—low mechanical resistance against dendrite growth [2]. However, these materials form excellent contact impedance.

The inorganic (ceramic) electrolytes have very good ionic conductivity, occasionally comparable to the conductivity of the liquid organic electrolytes [5,6], good resistance to dendrite growth and better thermal stability than polymer conductors [7]; however, they tend to form high impedance contacts. There are approximately five categories of ceramic electrolytes: perovskites, super ionic conductors (LiSICON, NaSICON), sulphates and salts.

The hybrid (or composite) electrolytes contain a blend of the other two types (polymer and ceramic). Either phase can be the main constituent. There are two approaches ceramic in polymer and, respectively, polymer in ceramic, depending on which phase is present with higher concentration. The composite materials merge the most important characteristics of the materials from within their composition, i.e., they have the high ionic conductivity of the solid-state electrolytes and the favourable interface characteristics of the organic electrolytes. Composites have an ionic conductivity in the order of 10^−5^ to 10^−3^ S/cm at room temperature. This conductivity value is comparable to the ionic conductivity of the liquid organic electrolytes currently employed in Li batteries (approximately~10^−2^ S/cm [6]). Table 2 shows a few examples of composite electrolytes and their respective conductivities.

Another very promising strategy is to construct hybrid electrolytes which consist of a mixture between solid-state electrolytes (SSE) and conventional liquid electrolytes (LE). These are quasi-solid-state electrolytes, and they achieve a compromise between safety and performance. With these materials the LE compensates for the shortcomings in terms of ionic conductivity of the SSE, but, at the same time, the amount of LE is not sufficient to cause any safety concerns [8].

**Table 2:** Ionic conductivity of various composite electrolytes [9,10,11].

Type of Material	Composition	Ionic Conductivity (S/cm)	Ref.
polymer—inert material	LiAlO_2_—PEO ^1^—LiClO_4_	10^−4^ (at 60 °C)	[12]
	TiO_2_—PEO—LiClO_4_	10^−5^ (at 30 °C)	[13]
	Al_2_O_3_—PEO—LiClO_4_	10^−2^ (60 °C)	[14]
	SiO_2_—Al_2_O_3_—PVDF-HFP ^2^-LiPF_3_(CF_3_CF_2_)_3_	10^−3^ (25 °C)	[15]
super ionic conductor—polymer	Li_5_La_3_Zr_2_O_12_—PEO—LiClO_4_	4.42 × 10^−4^ (55 °C)	[16]
	Li_2.5_Al_0.5_Ge_1.5_(PO_4_)_3_—PEO—LiClO_4_	2.6 × 10^−4^ (55 °C)	[17]
	Li_10_GeP_2_S_12_—PEO—LiTFSI ^3^	10^−5^ (25 °C)	[18]
	Li_6.2_Ga_0.3_La_2.95_Rb_0.05_Zr_2_O_12_—PVDF—LiTFSI	1.62 × 10^−3^ (25 °C)	[19]
perovskite—polymer	Li_0.33_La_0.557_TiO_3_ nanowires—PAN-LiClO_4_	2.4 × 10^−4^ (25 °C)	[20]
solid polymer—ionic liquid	LiTFSI—EMImTFSI ^4^—PEO	10^−2^	[21]
metal-organic framework	Zn_4_O(BDC)_3_^5^—PEO	3.16 × 10^−5^	[22]
solid polymer—cellulose	PEO—LiClO_4_—cellulose	10^−4^ (25 °C)	[23]

^1^ PEO = PEG = polyethylene oxide = polyethylene glycol; ^2^ PVDF-HFP = poly (vinylidene difluoride-co-hexafluoropropylene); ^3^ LiTFSI = lithium bis (trifluoromethanesulfonyl)imide; ^4^ EMImTFSI = 1-Ethyl-3-methylimidazolium bis (trifluoromethylsulfonyl)imide; ^5^ BDC = 1,4 benzodicarboxilate.

**Figure 1:**
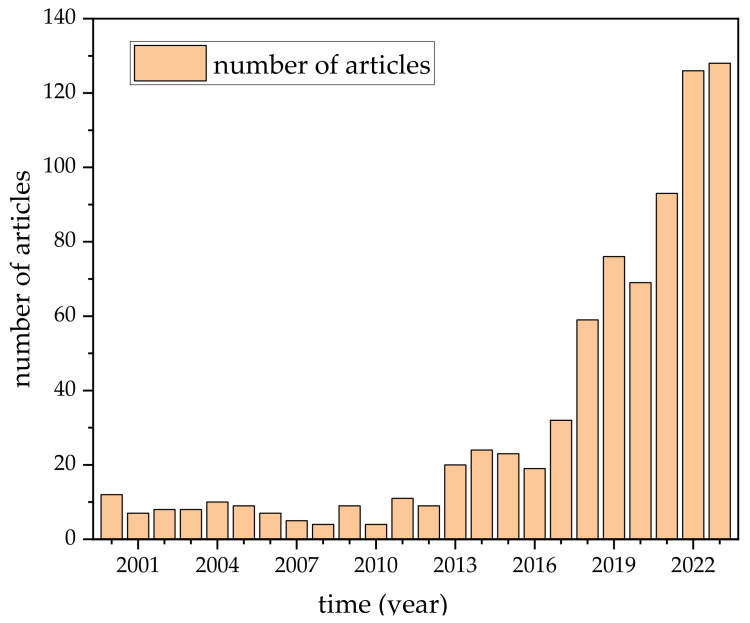
Number of publications on LLTO.

Lithium lanthanum titanates (LLTO) are a promising class of perovskite ionic conductors. Extensive research effort has been invested into the study of this material. This is evidenced by the large number of articles published on the LLTO topic over the past few years (Figure 1).

### 1.1. Applications

LLTO presents a high ionic conductivity, and it is stable in air. These properties make it a very attractive material for usage as a solid electrolyte. The rapid development of LLTO must be seen in the greater context of the evolution of lithium batteries.

Some recent, remarkable achievements include the development of new electrodes and new electrolytes [24,25,26]. Wu et al. [24] have successfully synthesised a new ternary eutectic electrolyte (with bis-trifluoromethanesulfonyl-imide, butyrolactam (BL) and succinonitrile (SN)) and achieved metallic Li/LiFePO_4_ batteries with excellent properties—dendrite free Li plating, 90% capacity retention after 500 cycles at 2 C and 99.8% coulombic efficiency. Sun et al. [25] studied a new class of anodes, namely the SiO_x_/C type which promise to achieve great capacities in the order of 2400 mAh/g. Liu et al. [26] used inorganic tubular fillers—rich in lone-pair electrons, to improve the ionic conductivity of a PEO-based composite electrolyte. The role of the inorganic tubular fillers is twofold. The fillers reduce the crystallinity of the polymer and also contribute themselves directly to the transfer of the Li ions. Other researchers are aiming at improving performance and lowering production cost. For example, Zhou et al. prepared, for the first time, a new anode material with the composition Li_4_Ti_5_O_12_ [27]. This new material is also used in the composition of the electrolyte. The electrolyte is a composite containing Li_4_Ti_5_O_12_ as the ceramic phase and poly (vinylidene fluoride) as the organic phase. The ionic conductivity achieved by the electrolyte is 2.87 × 10^−4^ S/cm at 35 °C. It was used for the fabrication of metallic Li—LiFePO_4_ cells which achieve a capacity of 150 mAh/g and have excellent capacity retention even under high discharge rates (119 mAh/g after 400 cycles at 5C) [27].

As it will be shown later, one of the limitations of LLTO in the usage as an electrolyte for metallic Li batteries is the instability of the material at the contact with metallic lithium. Some very recent works were focused on solving exactly this problem, i.e., developing a new composite electrolyte with enhanced stability. Bohao et al. [28] synthesised a composite electrolyte based on Li_0.95_Na_0.05_FePO_4_. The conductivity of the composite electrolyte reached 3.58 × 10^−4^ S/cm at 25 °C. The authors assembled a Li|composite electrolyte|LiFePO_4_ battery, which shows excellent capacity retention of 96.5% (or 151.5 mAh/g) after 500 cycles [28]. Of course, these are only few examples meant to define the context of LLTO research. Mazzapioda et al. prepared an excellent review article which highlights the challenges and the proposed solutions for obtaining solid-state electrolytes optimised for usage in metallic Li batteries [8]. Specifically, the review highlights the importance of quasi-solid-state electrolytes—these materials consist of conventional solid-state electrolytes to which ionic liquids are added for the purpose of improving the conductivity of the base solid-state electrolyte and the wetting at the metallic Li/solid electrolyte interface [8].

It should be noted that the possible applications for the LLTO are not limited to solid electrolytes. Further applications are envisaged for this material. Some of them are presented below. Details on the exact role of the material for these applications are also discussed on subsequent chapters (namely chapters 5 and 6).

#### 1.1.1. Li Extraction

Because LLTO permeates Li ions with ease, it can also be employed in Li recovery from waste batteries [29]. Here, Li is separated by electrodialysis. LLTO forms an ion separation membrane that is highly Li selective due to the intrinsic Li-ion conduction properties of the material. Extraction speeds of 3.5 mg/h were experimentally achieved from an alkaline solution containing LiOH, NaOH and KOH [29].

#### 1.1.2. Battery Electrode

The material presents high electronic conductivity at high temperature and/or high lithium concentration, which is a disadvantage for electrolyte applications, but is desirable for usage as a battery anode or a battery cathode [30,31]. As an electrode material, the increased electronic conductivity of the material could improve the contact with the current collectors and could lower the internal resistance of the battery. Moreover, the increased ionic conductivity could help to increase the Li-ions’ diffusion distance through the cathode (when LLTO is used as a “doping” material). LLTO can also present Li storage properties under some specific conditions. LLTO reacts with metallic Li below 1.5 V. The Li ions firstly occupy the vacant A sites, then they seem to occupy the 3c crystallographic positions while reducing Ti^4+^ to Ti^3+^. These mechanisms were tested, and a reversible capacity of 145 mAh/g was achieved [31].

#### 1.1.3. Sensors

The material also presents the property of H^+^/Li^+^ exchange and can therefore be used in a pH measurement device [32]. The sensing mechanism is observed in electrical impedance spectroscopy (EIS) experiments. It seems to be correlated to some reaction that occurs at the grain boundaries [32]. LLTO can also be used in a H_2_S sensor [33,34]. The H_2_S sensing mechanism is the following. LLTO is brought into contact with a semiconductor such as In_2_O_3_ [33] or SnO_2_ [34], forming a junction. In air, oxygen is absorbed on the surface of the material where it becomes ionised as O^−^ or O^2−^. The presence of the ionic oxygen species on the surface of LLTO has a twofold effect. Firstly, it generates an electron depleted layer at the LLTO–semiconductor junction. This increases the electrical resistance of the junction. Then, when H_2_S reaches the surface of the sensor, the surplus oxygen ions in the material causes the decomposition of H_2_S into SO_2_ and H_2_O with the release of electrons. (3e^−^ are released for the H_2_S reaction with 3O^−^, respectively, 6e^−^ for the reaction with 3O^2−^). This restores the electron density at the interface and the electrical resistance of the sensor decreases.

#### 1.1.4. Electronic Devices

Other uses include resistive switching devices (memristors) [35], or doping material for optoelectronic devices [36] and charge dissipation components in high voltage direct current (HVDC) cable insulators [37]. As a resistive switching device LLTO is incorporated in a Pt/LLTO/Pt thin film structure which presents self-rectifying characteristics (i.e., the electrical resistance of the device depends on the direction and magnitude of the previous current that passed through it). The Li-ion migration and the presence of oxygen vacancies dictate the resistive switching of the device, triggering electron trapping and detrapping processes [35]. Schottky contacts are formed at the Pt/LLTO interfaces. Under positive bias, electrons are initially easily injected into the bulk LLTO and somehow seem to overcome the Schottky barrier at the opposite contact. In this configuration the oxygen vacancies favour trapping electrons. In the negative bias, the injected electrons now have to overcome the new Schottky barrier (while the oxygen vacancies release the previously trapped electrons) [35].

LLTO doping in (K,Na)NbO_3_ systems (KNN), specifically in the (1 − x) (0.94K_0.51_Na_0.5_NbO_3_ − 0.06SrZrO_3_) − xLi_0.5_La_0.5_TiO_3_ ceramics, seems to increase the optical transmittance of the sample, and this allows observations of photosensitive resistance effects [36]. The defects in KNN apparently induce various energy levels in the band gap which favour charge carrier separation (leading to high conductivity) under exposure to strong light in the visible spectrum [18].

The insulation layer on high voltage DC cables needs to be very high, but this could also lead to the accumulation of space charge in the insulator layer, which itself causes a nonuniform electric field distribution and can lead to the insulation failure [37]. To prevent this, HVDC cables are equipped with a semiconductive layer which covers the conductive core and helps to uniform the electric field. Semiconductors, though, present the positive temperature coefficient (PTC) effect, which refers to a sharp increase in resistivity with temperature. It was shown that addition of LLTO to the semiconductive shielding can reduce the PTC effect.

### 1.2. Introduction to Perovskites

Perovskite oxides are compounds of the ABO_3_ type where A and B are cations. The best-known perovskite material, which gives the name to the class, is the CaTiO_3_ mineral. The two most commonly encountered types of perovskites are the “2-4” type and the “3-3” type. This classification is performed based on the oxidation states of the A- and B-site cations. In the former class the oxidation state of the A-site cation is 2+ and correspondingly the oxidation state of the B-site cation is 4+. Examples of this class include materials well known materials such as BaTiO_3_, SrTiO_3_, PbTiO_3_ and PbVO_3_ [38,39,40]. In the latter class, the valence of both A and B cations is 3+. Examples of these materials include BiFeO_3_, BiCrO_3_ and BiMnO_3_ [41,42,43,44,45].

Perovskites are very widely spread and have many applications in electronics (capacitors, piezoelectric transducers, computer memory devices), photovoltaic panel and fuel cells, to name just a few. They are also widespread in the field of Li batteries where they are used for the role of anodes, cathodes and electrolytes [46,47,48,49,50].

Lithium lanthanum titanates are perovskite oxides with the general composition Li_3x_La_2/3−x_□_1/3−2x_TiO_3_, where “□” denotes A-site vacancies. The electric charge of the oxygen ion is −2e. This means that the sum of the positive charges of the two cations must be +6e (assuming no oxygen vacancies) to maintain charge neutrality. For Li_3x_La_2/3−x_□_1/3−2x_TiO_3_ the B-site cation is Ti. The A site is occupied by La and Li. For x < 0.16 vacancies appear on the A site. The oxidation state of Ti in LLTO is 4+, but the oxidation state of La is 3+. Unlike the case of the two typical perovskite types mentioned above (2-4 and 3-3), this configuration leaves a charge of −2e that somehow needs to be compensated by a mixture of 3+ (La) and 1+ (Li) ions. It is obvious that in the simple case of no Li content the A site cannot be fully occupied while maintaining charge neutrality. The maximum occupancy for La^3+^ is therefore 2/3. As a consequence, the number of vacancies must be 1/3. This sets the lower boundaries for the substitution.

The oxidation state of Li is 1+ which implies that for every La^3+^ ion removed from the crystallographic structure, 3 Li^+^ must be added. It is obvious then that by this mechanism, two former vacancies must be now occupied. When x = 0.16 all vacancies are occupied. This sets the upper boundary for the substitution.

A slightly more mathematical description of the process shows that the stoichiometric coefficients for the two A-site ions are 2/3 − x for La and 3x for Li. The number of available vacancies must be, according to Equation (1):

(1) $$n_{vacancies}=1-\left[\left(\frac{2}{3}-x\right)+\left(3x\right)\right]=\frac{1}{3}-2x$$

## 2. Synthesis

A number of synthesis techniques have been employed for the preparation of the LLTO compounds. These can be divided on two subcategories: bulk material synthesis and nanostructured material synthesis. The first category contains primarily two methods: sol-gel method and the solid-state reaction method. Nanostructured materials are obtained by thin film deposition techniques and by electrospinning.

### 2.1. Sol-Gel Synthesis

The sol-gel method consists in dissolving organic or inorganic compounds of the metallic ions that form the final product, (for LLTO Li, La and Ti) and the dissociation of the metallic ions in a liquid solution. An organic compound (chelation agent) which forms chemical bonds with the metallic ions is added to the solution. The metallic ions then become bound to the chelation agent in the sought stoichiometry. The solution thus obtained is slowly evaporated and transformed into a gel. The gel is dried and undergoes calcination and sintering processes.

A good example of this approach is recently (2022) found in the works of Diktanaitė et al. [51]. They successfully synthesized Li_0.35_La_0.55_TiO_3_ using for starting materials LiNO_3_, La_2_O_3_, metallic Ti powder and HCl. The chelation agent they used was the tartaric acid (C_4_H_6_O_6_). The Ti^4+^ ion was obtained by the reaction between the metallic Ti and HCl and the formation of the [Ti(OH_2_)_6_]^3+^ ionic species. A similar approach was conducted earlier (published 2019) by Kežionis [30] when the precursors to the synthesis of Li_0.35_La_0.55_TiO_3_ were again LiNO_3_, La_2_O_3_ and metallic Ti. HCl was used as a solvent for Ti and La_2_O_3_ and tartaric acid was used as the chelation agent. Other research groups used different reagents. Tetrabutyl titanate, the chemical composition of the substance is Ti[OCH(CH_3_)_2_]_4_, is reported as a source of Ti ions [52,53,54,55]. The sources for Li and La are usually LiNO_3_ and La(NO_3_)_8_ [32,54,55]. The chelating agent is very often citric acid (C_6_H_8_O_7_ in either anhydrous or hydrated form) or tartaric acid (C_4_H_6_O_6_) [1,6,30,51,53]. Sometimes the starting compounds are heated to remove any traces of water that might influence the weighing errors. Moreover, the Li source compounds (LiNO_3_) is added with approximately 7 to 10% weight excess to the solution in order to compensate for the evaporation of the compound [30,51,56,57].

The reaction conditions are discussed below. Diktanaitė et al. [51] produced the gel by evaporating the liquid solution at 90 °C. The gel was subsequently dried at 120 °C. The drying stage was followed by a set of calcination treatments at temperatures ranging from 800 to 1100 °C to form the final product (LLTO). The LLTO powder is then reground, pressed into pellets and sintered at 1250 °C. Kezionis [30] produced the gel, then dried it at 120 °C and calcinated the powder at 1000 °C for 5 h. The sintering temperature was approximately 1250 °C. Further examples of synthesis conditions are provided in Table 3. Generally, the temperature ranges employed are the following: evaporation 55–95 °C, drying 100–150 °C, calcination at 350–1000 °C (most authors report calcination temperatures in a narrower range 400–800 °C [1,31,32,51,52,53,54]), sintering between 900 and 1350 °C (most publications between 1000 and 1250 °C [30,32,51,52,53,56]).

### 2.2. Solid State Reaction

The solid-state reaction method consists in mixing stoichiometric quantities of oxides and/or carbonates of the metallic ions that are required in the final product, pressing the powder into pellets and calcinating the mixture at temperatures in the order of 850–1200 °C. Reaction constants are generally low for solid state reactions therefore this method requires elevated temperatures (and possibly high pressure) and long reaction times. The reaction mechanism is slow for the following reason: in order to obtain a material with the chemical composition AB from two reagents A, respectively, B, it is necessary to remove atoms from the crystal lattice of A and to transfer them into the lattice of material B. The situation is symmetrical from the perspective of B. The reaction occurs initially at the interface between the crystallites of the two reagents. The AB compound (formed at the interface) therefore behaves as a barrier to the continuation of the reaction. In order for the reaction to continue A ions not only have to be removed from the lattice of material A, but also have to be transferred through the crystal structure of the AB material before they can be accommodated into the crystal lattice of material B. Sometimes it is required to stop the heat treatment and regrind the powder, before the reaction can be continued [58].

For the synthesis of the LLTO material, through the solid-state reaction process, the following reagents are commonly used: Li_2_CO_3_, La_2_O_3_ and, respectively, TiO_2_. Lanthanum oxide, La_2_O_3_, is hygroscopic and, when exposed to air, decomposes reversibly to lanthanum hydroxide, La(OH)_3_. Many authors employ a heat treatment at approximately 1000 °C for up to 12 h to the lanthanum oxide before weighing [7,59]. Moreover, similarly to the situation of the sol-gel method, excess Li_2_CO_3_ is used to compensate the material losses through evaporation. Li evaporation is a major concern for the synthesis of LLTO. One technique to also limit the evaporation, and the potential reactions of the powder with the crucible, is to shield the LLTO pellet with sacrificial powder of the same composition [6].

Kazumasa et al. [60] obtained materials from the LLTO class, namely Li_0.16_La_0.62_TiO_3_ and Li_0.33_La_0.56_TiO_3_ (x ≈ 0.05, respectively, x = 0.11) starting from two intermediary compounds, La_2_Ti_2_O_7_ and Li_4_Ti_5_O_12_, according to the following reaction (2):

(2) $${La}_{2}{Ti}_{2}O_{7}+{Li}_{4}{Ti}_{5}O_{12}\overset{yields}{\to }LLTO$$

Li_4_Ti_5_O_12_ is acquired as a precursor. La_2_Ti_2_O_7_ is prepared by calcination of a mixture of La_2_(CO_3_)_3_, TiO_2_ and KCl at 1200 °C for 8 h.

Generally [5,7,57,59,61,62,63,64,65,66,67,68,69,70,71,72,73,74] reaction temperatures are set in the following ranges: calcination at 650–1300 °C (mostly at 800 °C for anywhere between 2 to 8 h dwell time) and sintering at 950–1450 °C with dwell times ranging from 2 to 16 h. More often the sintering conditions are found in a narrower range, between 1200 and 1350 °C for 6 to 10 h. Further, specific examples of solid-state reaction conditions are presented in Table 4.

### 2.3. Thin Films

Thin film deposition techniques employed for LLTO synthesis include, e-beam evaporation, spin coating, dip coating, pulsed laser deposition (PLD) and magnetron sputtering [35,67,75,76,77,78,79]. The review will only remind the basic working principle of PLD and magnetron sputtering. An excellent and extensive body of literature exists on these topics [58,80]. The two techniques are typically using a target with a chemical composition close (sometimes identical) to the composition of the deposited film. Atoms are removed from the target and transported onto a substrate where they re-arrange and grow into the film. Both techniques require high vacuum. With PLD the target ions are removed by laser ablation. Magnetron sputtering uses an argon plasma to achieve the removal of the target ions. The Ar ions are accelerated towards the target and used for bombarding the target surface, which removes target ions. The plasma can be created by ionizing the Ar gas in either DC (if the target is conductive) or, as it is more often the case, AC (this can be applied to either conductive or insulating targets). Usually, the AC case is referred as radio-frequency magnetron sputtering, the typical frequency is 13.56 MHz [81]. The deposition is achieved by careful control of the many deposition parameters such as RF power, plasma composition (argon or argon/oxygen mixtures), vacuum pressure, distance between target and substrate, substrate temperature, target and substrate composition. A quick overview of deposition parameters used for the growth of LLTO thin films is provided in Table 5.

### 2.4. Electrospinning

Electrospinning is a technique used for generating nanowires [56]. It uses the property of a high strength electric field to cause the formation of a thin liquid jet. Without insisting on the details, the process is as follows: LLTO precursors are mixed/dissolved within some liquid polymer. The obtained mixture is then loaded onto a syringe with the needle connected to some high potential, typically in the range of 7 to 20 kV [33,56]. Since the electric filed intensity is inversely proportional to the distance, on sharp points (such as the tip of the needle, where the tip radius is small), the strength of the electric field is sufficiently high to cause by electrostatic repulsion the deformation of the liquid droplet (the formation of a Taylor cone) and the extrusion of the polymer mix as a narrow stream. This stream is then collected onto a surface where it forms a thin foil. The membrane thus obtained is then dried, calcinated and sintered to form LLTO nanowires.

### 2.5. Comparison between the Typical Methods of Synthesis for Bulk LLTO Samples

The most common synthesis methods for bulk LLTO samples appear to be sol-gel (including modified sol-gel variations) and solid-state reaction. On both methods some preparation of the reagents is observed. Heat treatments for La_2_O_3_ and TiO_2_ are carried out at relatively high temperatures (800–1200 °C) for long durations (10–12 h) [1,6,57,74]. Generally, the solid-state reaction method (compared to the sol-gel method) requires higher temperatures longer dwell times and multiple heat treatments interrupted by intermediary grinding steps [61,82]. Single phase LLTO samples are typically not obtained as a result of sol-gel synthesis. These types of samples often contain small amounts of secondary reaction products, such as La_2_Ti_2_O_7_ [51], Li_2_TiO_3_ [52,74] or various mixtures of LLTO type compounds, such as Li_0.35_La_0.55_TiO_3_–Li_0.125_La_0.625_TiO_3_ [54]. The concentration of impurities decreases with the increase in the synthesis temperature [32].

**Figure 2:**
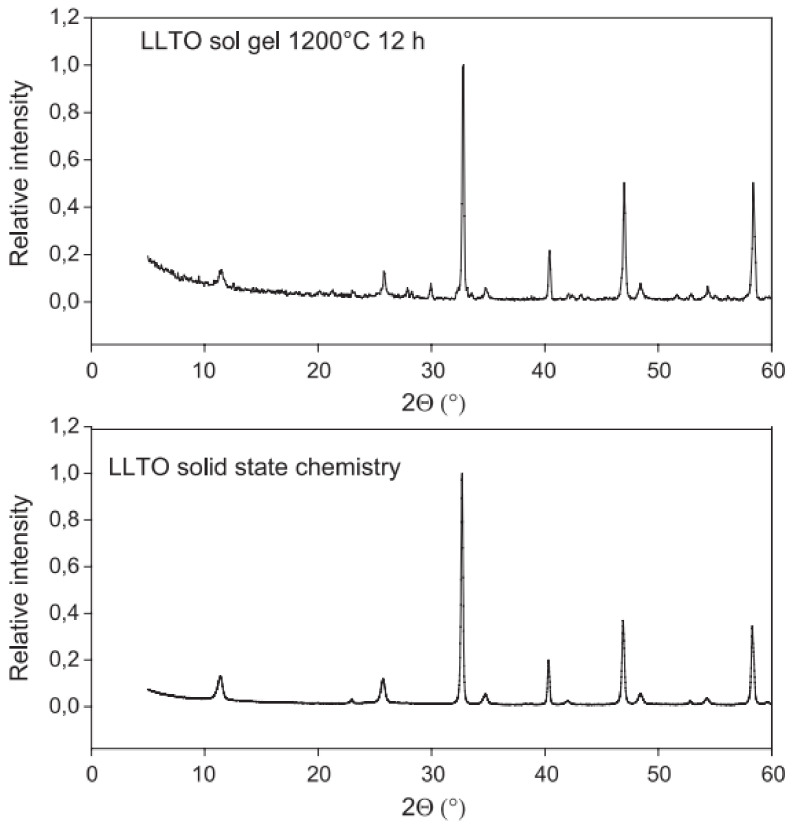
X-ray diffraction pattern for LLTO samples obtained through the sol-gel process and, respectively, solid-state reaction [32]. Reprinted with permission. Copyright 2005 Solid State Ionics—Elsevier.

**Figure 3:**
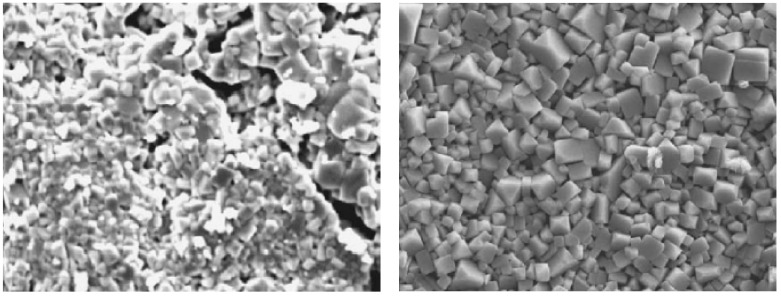
SEM imaging for LLTO samples synthesised through sol-gel and, respectively, solid-state reaction [32]. Reprinted with permission. Copyright 2005 Solid State Ionics—Elsevier.

Solid-state reaction tends to produce cleaner samples. It should, however, be noted that La_2_Ti_2_O_7_ is also observed as a secondary reaction product during solid-state reaction experiments [32]. Figure 2 shows XRD patterns corresponding to LLTO samples obtained through both methods, according to the works of Bohnké et al. [32]. It can be easily observed on the figure that in the case of the solid-state reaction the quantity of impurities in the final product is lower than the quantity of impurities formed in the case of the sol-gel method. Samples obtained by solid state reaction tend to contain larger crystallites (in the order of micrometres) compared to the crystallites observed when samples are prepared through sol-gel (100–500 nm) [75]. The same observation is confirmed by SEM, as shown in Figure 3.

The comparison between the two synthesis methods in terms of properties of the LLTO final product was extensively studied by Bohnké et al. [32,82]. The authors used LLTO for pH sensor applications. With this application, the sensitivity of the device depends on the distribution of grain sizes. Similar sensitivities are obtained with samples prepared by either method. However, the synthesis using solid-state reaction is less cost-effective. The sample preparation time is 5 days for the solid-state reaction method and 5 h for a modified sol-gel method. Moreover, the yield is greater for the sol-gel method [82].

## 3. Structural Properties of LLTO

LLTO can be obtained under different crystallographic structures (cubic, tetragonal [57], orthorhombic [63], hexagonal [56]) depending on the composition (Li/La ratio) and synthesis conditions [57,63].

The discovery of LLTO is credited to Inaguma [61,73,83] Latie [84] and Belous [85]. The first observations were that the structure of Li_0.34(1)_La_0.5(1)_TiO_2.94(2)_ (where the metallic composition is verified by ICP and the oxygen content is calculated for charge neutrality) is cubic and that the material presents ordering of the Li^+^, respectively, La^3+^, ions leading to the formation of a superstructure. In the superstructure model, LLTO crystallizes with the space group P4/mmm and the lattice parameters a = 3.8710(2) Å and c = 2a [61]. The superstructure consists of alternate La-rich, respectively, La-poor (Li and vacancies rich layers). The ordering of these layers can be understood as double stacking of the La-rich layers along the c axis. Two La-rich layers are thus found at a distance equal to twice the length of the unit cell. The interplanar distance of c = 2a produces a characteristic X-Ray powder diffraction peak defined by fractional Miller indices, namely (0, 0, ½). This peak appears at a diffraction angle of approximately 2ϴ = 11° and is used as an indicator of the presence of the superstructure [61]. It is apparent that the Li ions do not directly occupy the vacant La sites. Instead, it has been suggested that the Li cations could occupy positions off-centre next to the La vacancies and positions defined by oxygen square windows [86].

These were the first studies on the material. It was later revealed that (as already mentioned) the phase diagram of LLTO is rather complex. The various crystallographic systems in which LLTO can be found and the corresponding composition/sintering parameters are tentatively reviewed in Table 6.

Subsequent studies on the structure of the material revealed that Li_0.5_La_0.5_TiO_3_ sintered at 1350 °C is tetragonal [87]. The space group is P4/mmm, as determined by XRD and Raman spectroscopy. The lattice constants are a = 3.6 Å and c = 7.2 Å, observed by HRTEM [87]. A similar observation was reported by Abhilash et al. [88]. Here Li_0.5_La_0.5_TiO_3_ was synthesized by the sol-gel method at a comparatively lower temperature of 900 °C. The crystallographic system obtained remains tetragonal, the space group remains P4/mmm; however, the lattice constants are larger at a = 3.93 Å and c = 7.86 Å.

Li_0.29_La_0.57_TiO_3_ sintered at 1400–1460 °C is orthorhombic [63] with the space group Cmmm and the lattice parameters a = 7.737(1) Å, b = 7.742(1) Å, c = 7.785(1) Å. Another report by Yang et al. [56] on a very similar material in terms of composition, namely Li_0.26_La_0.61_TiO_3_, with the composition determined by ICP, showed that the crystallization system is again tetragonal. The compound was obtained by Yang et al. for the first time by electrospinning. Due to the nature of the technique the calcination temperature required was low (1000 °C for 3h dwell time) compared to requirements for solid state reaction [56]. Yang [56] also mentions, citing other authors, that LLTO can be obtained under hexagonal systems [89]. For the main focus of the works, tetragonal LLTO obtained by electrospinning, the observed lattice parameters are a = 3.875 Å, respectively, c = 7.739 Å. These parameters are determined/confirmed by XRD, SAED and HRTEM [56].

**Figure 4:**
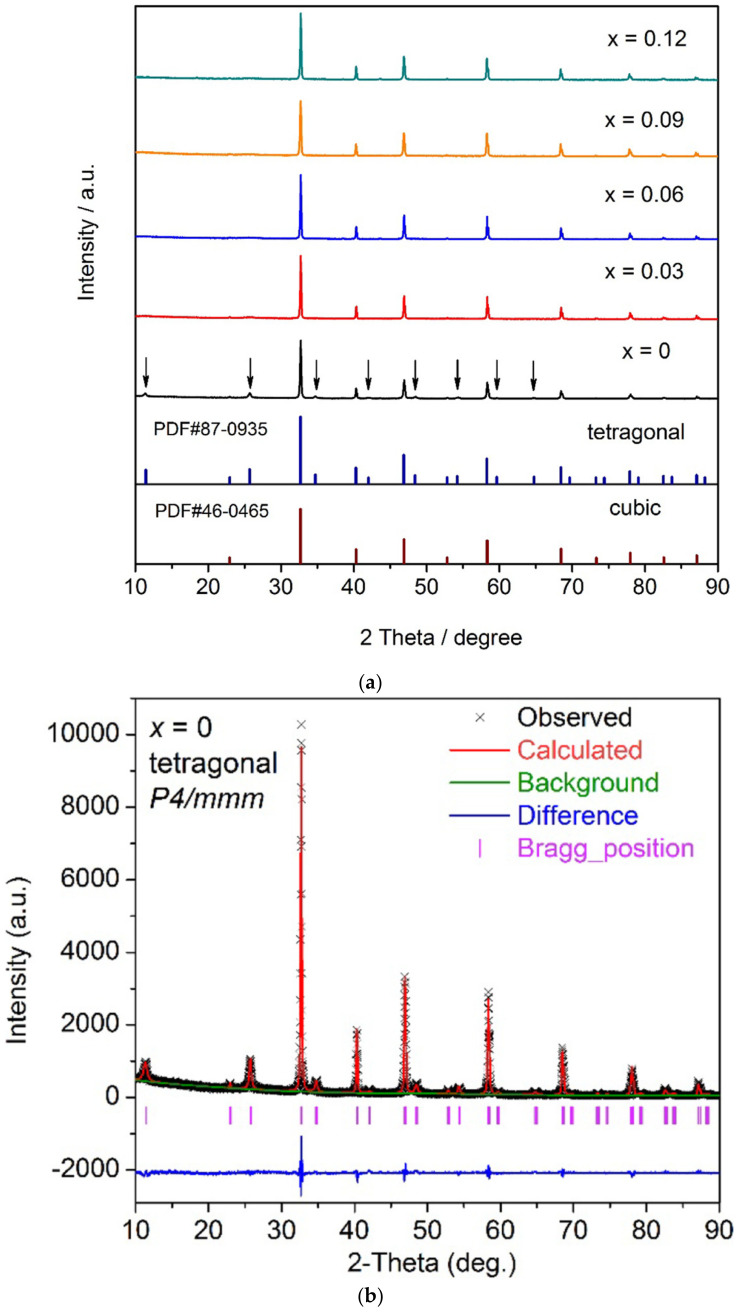
(**a**) XRD patterns for LLTO, according to reference [6]. Reprinted with permission. Copyright 2019 Solid State Ionics—Elsevier. (**b**) Rietveld refinement for LLTO, according to reference [6]. Reprinted with permission. Copyright 2019 Solid State Ionics—Elsevier.

Figure 4 shows the XRD patterns and Rietveld refinement for LLTO samples in which La is substituted with Sr [6]. The results are also relevant for pristine samples. Superstructure peaks of the pristine samples are identified by arrows. The Powder Diffraction File database (PDF) card identification numbers found by the authors as indicatives for the presence of the cubic and tetragonal phases respectively are also shown on the figure.

Xu [90] observed that both tetragonal and orthorhombic phases coexist within the nominal Li_0.33_La_0.55_TiO_3_ compound. The main phase contains Li_0.33_La_0.55_TiO_3_ is tetragonal and consists of large grains. Within the large grains, smaller ones are observed, corresponding to a Li-poor phase with the Li_0.18_La_0.6_TiO_3_ composition. The Li poor phase is orthorhombic. The results are obtained by HRTEM and electron diffraction.

Kazuhiro [65] used the ^7^Li isotope to accurately determine the position of the Li ions within the LLTO unit cell by time-of-flight neutron powder diffraction (TOF-NPD). ^7^Li was used because it has a much lower neutron absorption cross section (σ = 0.045 b) compared to naturally occurring Li (σ = 70.5 b). The composition of the material prepared by Kazuhiro [65] is ^7^Li_0.4_La_0.53_TiO_3_. The samples are prepared by solid state reaction, sintered at 1300 °C and then quenched in liquid N_2_ [65]. With this setup the LLTO phase is orthorhombic with the space group Cmmm. The results showed that the Li ions randomly occupy A sites within the perovskite cell.

It is perhaps somewhat complicated to form a clear image of the synthesis–composition–structure correlation by simply examining isolated cases; therefore, broader and more systematic studies of the LLTO system are the focus of the next examples discussed in this article.

Zhou [62] prepared LLTO samples with different Li compositions at 1250, 1300 and 1350 °C. Zhou showed that low Li compositions (3x = 0.16) yield orthorhombic structures for all the tested sintering temperatures. The orthorhombic phase is also obtained for higher Li concentrations (3x = 0.33 and 0.4) if the temperature is raised to 1300–1350 °C. If the Li content is relatively high (3x = 0.33, 3x = 0.4) and the sintering temperature is relatively low (1250–1300 °C) the obtained phase is tetragonal [62]. The orthorhombic phase is defined by the space group Pmmm and the tetragonal phase is characterised by the P4/mmm space group. The superstructure diffraction peak (0, 0, ½) is observed at 2θ = 11.5°. The intensity of the superstructure peak is decreasing with increasing Li concentration.

**Figure 5:**
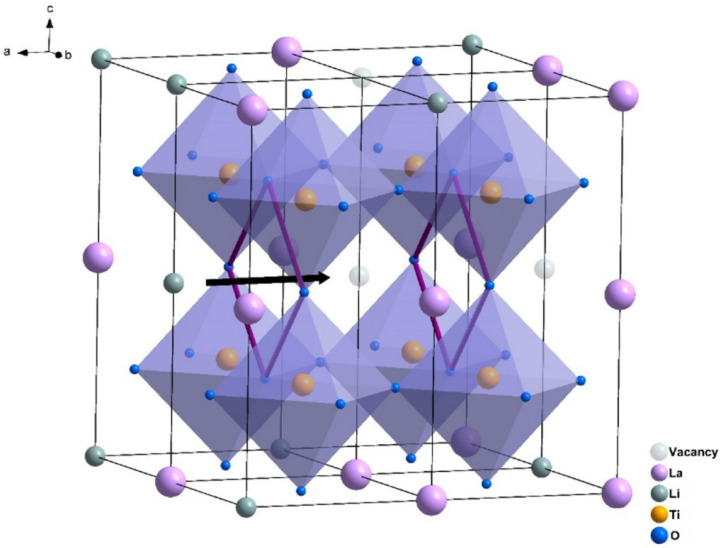
LLTO structure from reference [6]. Reprinted with permission. Copyright 2019 Solid State Ionics—Elsevier.

Diktanaitė [51] observed the formation of the tetragonal and orthorhombic phases as follows: for Li_0.3_La_0.567_TiO_3_ sintered between 800 and 1100 °C, the tetragonal phase is stabilized. Li_0.35_La_0.55_TiO_3_ on the other hand crystallizes in an orthorhombic system for sintering temperatures between 1100 and 1250 °C. The lattice constants vary little with the composition and the sintering temperature, at approximately a = 5.47–5.49 Å and c = 7.71–7.77 Å for the tetragonal phase, respectively, a = 7.743–7.746 Å, b = 7.742–7.734 Å, c = 7.737–7.739 Å for the orthorhombic phase [51]. The authors thus concluded that the Li/La ratio dictates the formation of the final crystalline phase [51]. They also studied the deformation of the unit cell between the two phases. LLTO presents the typical perovskite structure. The Ti ions are surrounded by an oxygen octahedron [88]. The TiO_6_ octahedra are corner-sharing [6,88]. Figure 5 shows the structure of Sr substitution LLTO [6] and highlights the conduction pathways. The Li/La ions are distributed on the corners of the unit cell [88]. The oxygen octahedron presents some distortion. The distortion is very little for the tetragonal phase and is larger in the case of the orthorhombic phase. The distortion degree is calculated based on the tolerance factor [51] and the interatomic distances within the TiO_6_ octahedra (obtained from Rietveld refinement). The tolerance factor is given by Equation below (3) and predicts the stability of a perovskite phase.

(3) $$t=\frac{R_{A}+R_{O}}{\sqrt{2}\left(R_{B}+R_{O}\right)}$$

R_A_ and R_B_ are the ionic radii of A and B cations and R_O_ is the oxygen ionic radius.

**Figure 6:**
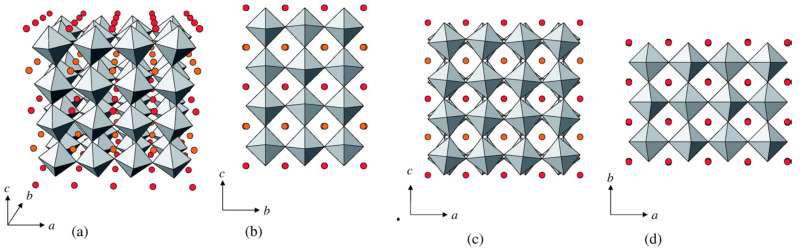
LLTO structure, TiO_6_ octahedra tilt and separation of La rich and La poor layers from reference [91], (**a**) 3D representation, (**b**–**d**) projections on planes (bc), (ac) and (ab), respectively. Reprinted with permission. Copyright 2006 Solid State Ionics—Elsevier.

**Figure 7:**
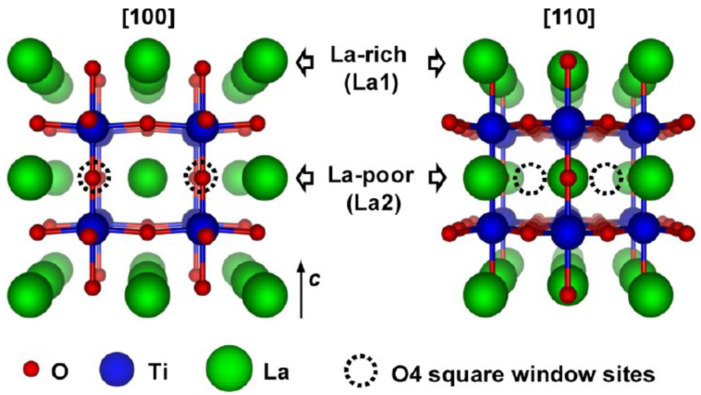
LLTO structure, separation of La layers [86]. Reprinted with permission. Copyright 2013 Chemistry of Materials—American Chemical Society.

Borštnar [57] highlighted the existence of two distinct LLTO phases, namely α-LLTO and β-LLTO, both having the same composition (x = 0.11) but different crystallographic structures. The former, α-LLTO is pseudocubic (Pm3m space group) and β-LLTO is tetragonal (P4/mmm space group). Quenching the material results in the formation of a cubic structure. Slow cooling on the other hand leads to the stabilization of the tetragonal phase [57]. The difference between the two phases consists in the distribution of the Li/La ions. The cubic phase, α-LLTO, is characterized by very small TiO_6_ octahedra tilts and randomly distributed Li^+^, La^3+^ and vacancies on the A site. On the contrary the tetragonal, β-LLTO, phase is defined by alternating La-rich and La-poor layers along the c axis which results in doubling the c axis [57]. Figure 6 highlights the tilting of the TiO_6_ octahedra (for pristine samples) Figure 7 shows the separation of La on La-rich and La-poor layers.

When the Li content is increased to x = 0.146–0.167 the tetragonal phase is stabilised with a = 3.87 Å and c = 2a. For lower Li content, x = 0.03–0.063 the system becomes orthorhombic, with the Pmmm space group and a = 3.864 Å, b = 3.875 Å and c = 7.786 Å.

## 4. Conduction Mechanism

The ionic conductivity of the LLTO material is closely related to the material structure. The corner sharing TiO_6_ octahedra form the pathways (also known as channels or bottlenecks) through which the Li ions can migrate. The ionic conductivity of the material depends on the concentration, the mobility and the total electrical charge of the charge carriers (here, Li^+^ ions). The correlation between these properties is described by an equation similar to the Equation below (4):

(4) σ=eVnμ

where σ is the conductivity, n is the concentration, µ is the mobility, V is the ion valence and e is the elementary charge.

### 4.1. Charge Carrier Concentration

**Figure 8:**
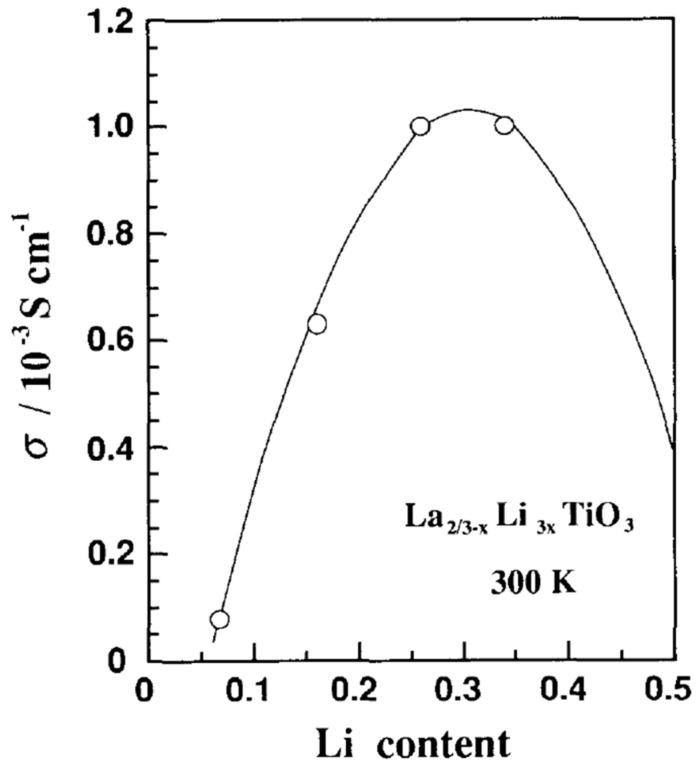
LLTO conductivity variation with the Li content, from reference [73]. Reprinted with permission. Copyright 1994 Solid State Ionics—Elsevier.

Increasing the Li ions concentration increases the ionic conductivity up to a point. If the Li concentration continues to rise, the ionic conductivity will decrease because the number of vacancies decreases. Thus, an optimum Li concentration is observed for x = 0.11 or Li_0.33_La_0.55_TiO_3_ [6,73,89,92]. Figure 8 shows the dependence of the ionic conductivity on the Li concentration for LLTO [73]. The discussion of this effect is resumed on the next section. Then the conductivity depends on the type of structure of the material. The cubic phase presents overall higher conductivity than the tetragonal phase. This is because in the tetragonal phase conductivity is anisotropic, meaning that ions migrate easier along the La-poor planes that through the La-rich planes. Conductivity is thus higher along the a and b directions than along the c direction. The cubic structure is obviously isotropic. Other factors influencing the conductivity are the presence of a domain structure within the material and the crystallite size. Conductivity is reduced at domain boundaries [62] and grain boundaries because the conduction pathways are interrupted. The complications on optimizing the ionic conductivity stem from the contradictory requirements that must be met. Larger crystallites reduce the number of grain boundaries and hence improve conductivity [57]. However, the growth of crystallites requires high temperatures and long dwelling times. This tends to favour the formation of the less conductive tetragonal phase and to enhance the loss of Li through evaporation. Similarly, quenching the sample favours the formation of the higher conductivity cubic phase, but, at the same time, leads to the formation of small size crystallites, respectively, increased number of grain boundaries, thus lowering conductivity. The review will examine below in more details the conduction mechanisms.

### 4.2. Grain Boundaries

The ionic conductivity properties of the material can be quantified by measuring a number of parameters using a corresponding set of investigation techniques. One approach is to construct actual working Li cells and measure their behaviour. This is particularly useful when the chemical stability of the electrolyte is tested against both electrodes or when the whole assembly needs to be thoroughly investigated. This is typically the goal of (Li ion) battery research. However, very often it is sufficient to construct symmetric cells by deposition of blocking electrodes directly on the electrolyte surface. Then conductivity is determined by electrical impedance spectroscopy (EIS). It should be mentioned that nuclear magnetic resonance (NMR) is also occasionally employed in the investigation of the Li-ion mobility (activation energy) and the results from NMR investigations are coupled to EIS results [84,93,94]. Examination of the Nyquist diagrams provides the means to separate the two components of conductivity, namely grain conductivity, or bulk conductivity, and grain boundary conductivity. Thus, the typical Nyquist diagrams contain two arcs and a straight section. The high frequency semicircle corresponds to the bulk conductivity, the low frequency semicircle corresponds to the grain boundary conductivity and the spike (straight section) is caused by the blocking electrodes themselves [62]. The values of the two conductivity components are decisive parameters and the article will be constantly referencing them. Then, ionic conductivity is a thermally activated process, following the Arrhenius law, or sometimes the Vogel–Fulcher–Tammann law [74,93]. The activation energy is another decisive parameter that will be monitored throughout the review. The change in the value of the activation energy could indicate a change in the Li^+^ conduction mechanism [74,93,95,96].

The aim of the experimentation is to understand the functioning of the conduction mechanisms and to improve the overall conductivity. As already mentioned, one of the main limiting factors is the grain boundary resistance which for LLTO can be up to two orders of magnitude higher than the bulk resistivity [70] (bulk conductivity can be as high as 10^−3^ S/cm while the grain boundary conductivity is in the order of 10^−5^ S/cm).

Thus, some authors [70,87,97,98,99] have attempted to mix the LLTO matrix with an inorganic amorphous material which should serve as a filler material bridging the conductivity gap between LLTO grains. Such filler materials include Al_2_O_3_, TiO_2_ and ZrO_2_ [97,98,99]. On all the cases, conductivity was improved, the effect being more pronounced for nanosized systems. Mei [87,97] succeeded in preparing a LLTO-SiO_2_ system. Neither SiO_2_ diffraction peaks nor diffraction peaks corresponding to other Si compounds were observed, thus it was concluded that the SiO_2_ fraction is indeed amorphous [97]. The Nyquist diagrams showed only one arc and one spike. The sample was modelled as follows: the bulk conductivity was defined by a simple resistor, the grain boundary component was described by a resistor connected in parallel to a capacitor and the blocking electrodes were defined as a simple capacitor. The best ionic conductivity was approximately 10^−4^ S/cm and it was achieved at 5 vol.% concentration of SiO_2_. The authors observed that the addition of SiO_2_ decreased the Li content of LLTO. It was concluded that SiO_2_ could have transformed into some Li silicate. More than 5 vol.% SiO_2_ reduced the Li concentration too much. Less SiO_2_ was observed to not improve grain boundary conductivity by any significant amount. Moreover, the authors noted that the thickening of the amorphous interface could be detrimental to conductivity [97]. In another study of the LLTO-SiO_2_ system Mei [87] noticed the anisotropy of the conductivity—conduction occurs along the Li/La plane but not perpendicular to the Li/La plane for a tetragonal system. The authors also observed that the conductivity is isotropic for a cubic system [87]. The material synthesized for this study was tetragonal [87], it showed the characteristic superstructure which indicates ordered distribution of La^3+^, Li^+^ and vacancies. The Nyquist plot again shows one arc and one line. It was observed that the grain boundary resistance decreases with the addition of SiO_2_, but, at the same time, the bulk resistance is increasing, possibly due to the amorphous Li silicate at the grain boundary which causes Li depletion within the grain itself [87]. Another interesting observation of the study is that the amorphous silica layer avoids conduction anisotropy [87].

Taiye [70] explored a different approach for eliminating the grain boundary effect, namely the synthesis of an amorphous LLTO-silica glass. The authors noted that pure LLTO cannot be obtained in an amorphous state by melting and rapid cooling due to the high cooling rates that would be required [70], but it is possible to mix LLTO with a silica-based glass. They prepared both types of samples, crystalline LLTO and amorphous LLTO. The crystalline sample showed all the diffraction peaks of Li_0.5_La_0.5_TiO_3_; however, no diffraction peaks were observed for the amorphous phase [70]. Two arcs are obtained for the crystalline sample; however, only one arc is present in the Nyquist plot of the glass composite. This observation could be understood by considering that the amorphous material has no grain boundaries. The ionic conductivity was improved from 4.73 × 10^−6^ S/cm for the crystalline perovskite to 1.3 × 10^−5^ S/cm for the glass composite [70]. It was also observed that the amorphous compound shows higher density and higher stability than the crystalline LLTO. It was concluded that the ionic carriers in the glass–perovskite system encounter a lower number of resistive areas as they travel through the material [70].

Borštnar [57] synthesised LLTO by gradually lowering the Ti content (increased La:Ti ratio), and thus triggered the growth of coarse grains, with dimensions up to 100 µm, and the formation of Ruddlesden–Popper structures with intercalated Li_2_La_2_Ti_3_O_10_ layers. The larger grain sizes are expected to reduce the density of grain boundaries. The authors prepared four sets of samples defined by the ratio of the starting oxides as y = 1 (nominal stoichiometric composition), 0.95, 0.925 and 0.9 representing La:Ti ratios of 0.56, 0.589, 0.605 and 0.622, respectively. Lowering the TiO_2_ content has the effect of shifting the point at which the densification process is completed to higher temperature values. Some secondary reaction products were identified as small amounts of TiO_2_ rutile and Li_2_Ti_3_O_7_. Grain sizes increase with decreasing y and increasing the sintering temperature [57]. For all samples the Nyquist spectra indicate the typical features (two semicircles and a straight line). The bulk ionic conductivity was approximately the same for all samples, in the order of magnitude of 10^−4^ S/cm. The quenched cubic phase presented slightly higher bulk conductivity than the slow cooled tetragonal phase [57]. Grain boundary conductivity was observed to be lower (6.25–9.18 × 10^−5^ S/cm) for the y = 1 and y = 0.95 samples. The corresponding Nyquist semicircle is well defined for these samples. As expected, the conductivity for the samples with the higher grain sizes (y = 0.925, y = 0.9) was higher than the grain boundary conductivity for the other samples. For the large grain samples, grain boundary conductivity was as high as 5.35 × 10^−4^ S/cm. Interestingly, among the high grain size samples, conductivity is higher for samples obtained by slow cooling, rather than quenching (4.86 × 10^−4^ S/cm for the slow cooled y = 0.9 sample and 2.63 × 10^−4^ S/cm for the corresponding, y = 0.9, quenched sample).

Xu [90] distinguishes between two types of grain boundaries at two different length scales: macroscopic grain boundaries in the order of micrometres and microscopic grain boundaries in the nanometric range. The authors prepared LLTO with the nominal composition of Li_0.33_La_0.56_TiO_3_ by sol-gel and the material is obtained in the tetragonal form. The material consists of large polycrystalline grains (grain sizes in the range of 5–10 µm). The larger grains are themselves formed from smaller, single crystallites of a few hundred nanometres. The two grain sizes lead to the formation of the two types of grain boundaries. A very interesting observation that should also be noted is that some of the macro-grain boundaries are actually void [90]. The authors determined by selected area electron diffraction (SAED) that some of the smaller grains consist of a different LLTO phase. These grains show a Li-poor composition corresponding to Li_0.18_La_0.6_TiO_3_ and crystallize in an orthorhombic system. The lattice parameters of the two phases are close. The authors hypothesised that the Li-poor phase forms by Li evaporation and rearrangement of the La ions. At any rate, the microscopic grain boundaries appear to be present with a higher concentration and therefore are assumed to be the main contributor to the grain boundary resistance. Contrary to the typical results, Xu [90] observed that the Li conductivity actually decreases with the increase in the size of the large grains, in spite of the decrease in the amount of grain boundaries. This is further evidence in support of the hypothesis that micrograin-boundaries, rather than the macrograin boundaries, are the main conduction limiting factor. The amount of the Li_0.18_La_0.6_TiO_3_ phase also constitutes a limiting factor. Conduction pathways are blocked by the La rich layers at the interface with the grains of the Li-poor, orthorhombic phase. The increased sintering time leads to the formation of larger grains, but also to the increase in the concentration of the Li-poor phase.

### 4.3. Domain Boundaries

**Figure 9:**
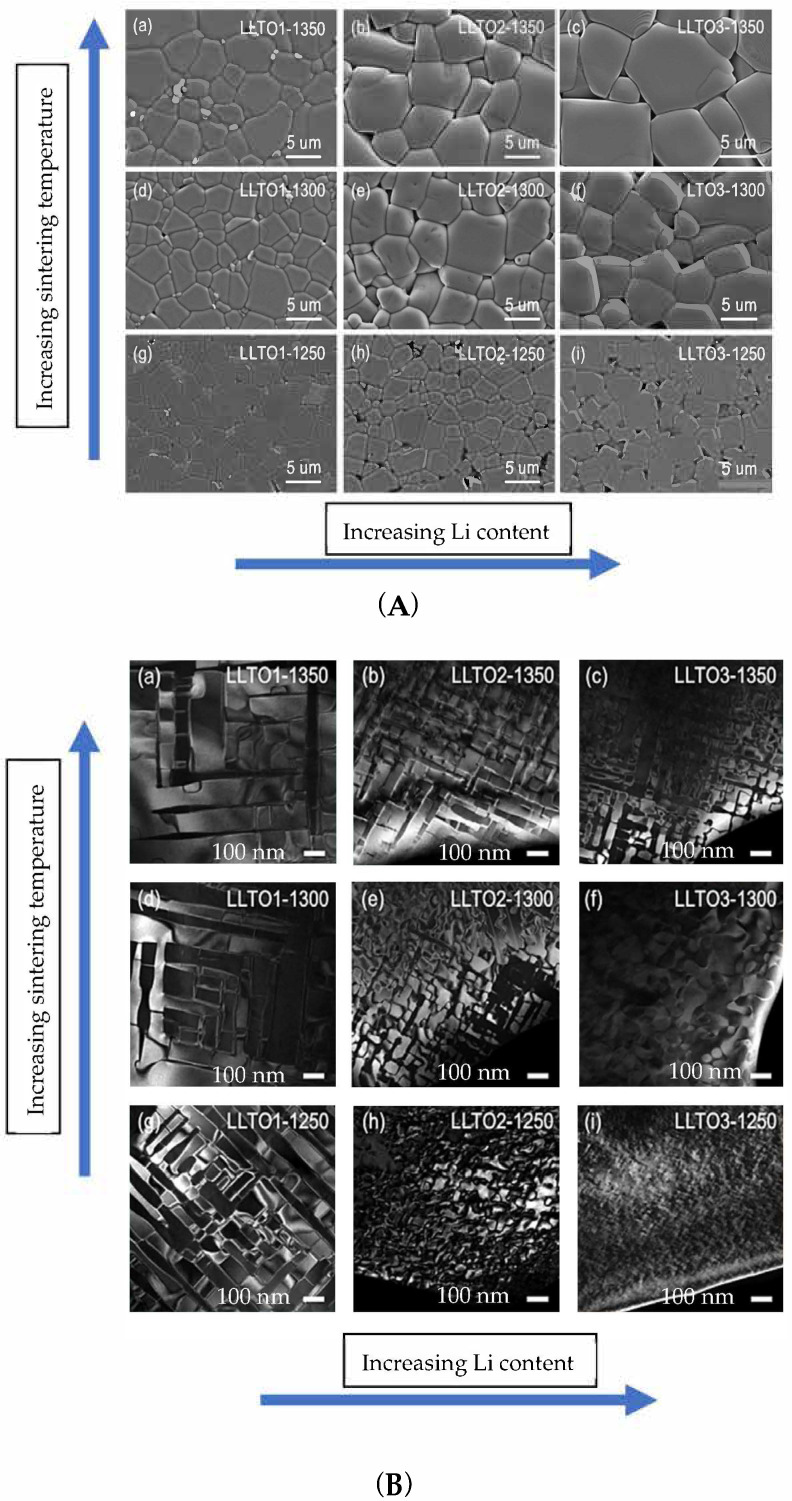
(**A**) Visualization of grain boundaries in LLTO from reference [62]. Reprinted with permission. Copyright 2022 Journal of Energy Chemistry—Elsevier. (**B**) Visualization of domain boundaries in LLTO from reference [62]. On both figures, the letters (**a**)–(**i**) indicate LLTO samples obtained at different temperatures and different Li contents. The variations of the two parameters are indicated by the blue arrows. Reprinted with permission. Copyright 2022 Journal of Energy Chemistry—Elsevier.

**Figure 10:**
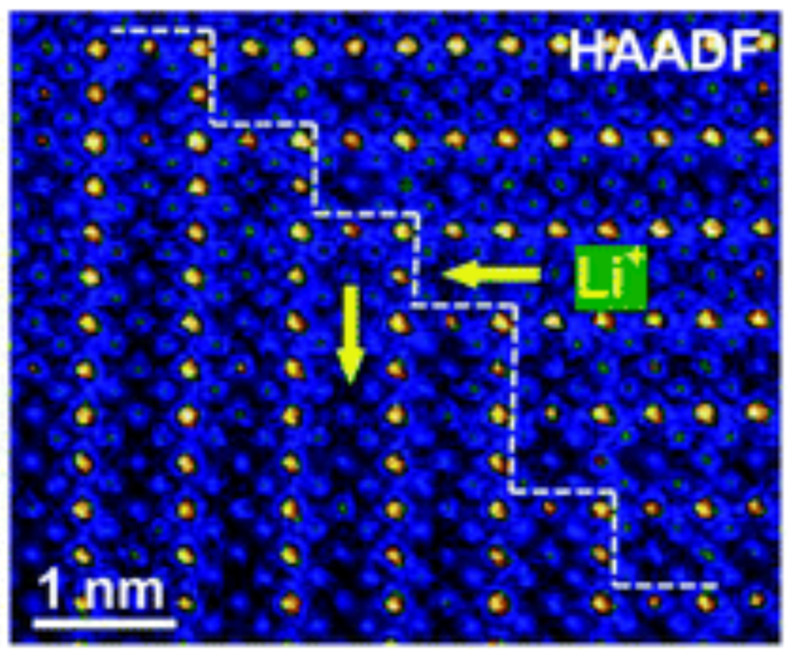
Visualization of domain boundaries in LLTO from reference [101]. Reprinted with permission. Copyright 2014 Journal of Materials Chemistry A—Royal Society of Chemistry.

LLTO presents an intricate domain boundary structure. The effects of the domain structure were investigated by many authors, for example: Moriwake in 2015 [100] and Zhou [62] in 2022. LLTO contains domain boundaries (DB) rotated by 90° [100,101]. The domains present La segregation at the interface and lattice strain. These effects act as barriers to Li conduction and therefore increase the resistance at the boundaries. The formation of these domains is intrinsic to LLTO. Both the domain boundary concentration and the domain boundary resistivity determine the Li conductivity [62]. Zhou [62] studied the correlation between domain boundary concentration and domain boundary resistivity. Nine samples were prepared consisting of three Li contents (3x = 0.16, 0.33 and 0.4) at three different temperatures (see previous section). Small amounts of TiO_2_ and La_2_Ti_2_O_7_, both apparently insulators for Li conduction, are sometimes seen as impurities. The authors observed that, depending on the crystal structure of the material (see previous section), two types of domains are formed. The samples which crystallize in the orthorhombic system tend to be characterized by elongated domains which produce straight domain boundaries. On the other hand, the samples that crystallize in the tetragonal system present mosaic type domains with curved domain boundaries. Contrary to the grain microstructure (which is shown by SEM results) domain boundary concentration increases with increasing Li content and decreasing sintering temperature. Sintering temperature or Li content can be used to tune crystal symmetry. The authors noted that decrease in the DB concentration results in increase in DB resistance due to increased lattice mismatch [62]. The high DB resistance, and not the DB concentration, is the main factor determining the conductivity. Samples with tetragonal structure and high concentration of curved DB have much larger conductivity than samples with orthorhombic structure with low concentration of straight DB. Activation energies are: for bulk E_A bulk_ = 0.309–0.337 eV, for grain boundaries E_A grain_ = 0.372–0.401 eV [62]. Figure 9 shows SEM images of LLTO grain boundaries and TEM images of LLTO domain boundaries, as reported in reference [62]. Figure 10 shows a colorised high-angle annular dark-field image (HAADF) of an LLTO sample, highlighting the presence of the domain boundaries [101].

### 4.4. Conductive Paths and Relaxation Mechanisms

Kazuhiro [65] used time of flight neutron powder diffraction and simulations by reverse Monte Carlo modelling and the bond valence sum approach to evidence the actual conduction pathways through LLTO (Li_0.4_La_0.53_TiO_3_). The crystallographic results were already discussed. The ionic conductivity of the sample was about 10^−3^ S/cm at 300 K [65]. The activation energy for the material was approximately 0.269 eV (26 kJ/mol) [65]. Because conduction properties are strongly dependent on the crystallographic properties of the material, some redundancy in the explanations is unavoidable, but, as previously discussed, the structure of LLTO consists of corner sharing TiO_6_ octahedra and alternating stacks of La-rich and La-poor layers along the c axis. The La ions occupy the 4i Wyckoff position in the La-rich layers and the 4j Wyckoff position in the La-poor layers [91,102]. Four TiO_6_ octahedra sharing four oxygen ions form a bottleneck [65]. Kazuhiro distinguished tree types of bottlenecks according to their cross-sectional area (S) as follows: type I for S = 0.07 nm^2^, type II for S = 0.08 nm^2^ and type III for S = 0.09 nm^2^ [65]. The authors determined by simulations that the type II bottleneck is the most accessible one for the Li-ion migrations and more than 70% of their quenched LLTO sample contains type II bottlenecks.

Šalkus [93] has investigated the temperature variation in complex conductivity, complex resistivity and complex dielectric permittivity for three samples of the LLTO system (x = 0.08, 0.1 and 0.12, which corresponds to Li_0.24_La_0.586_TiO_3_, Li_0.3_La_0.56_TiO_3_ and Li_0.36_La_0.546_TiO_3_). A non-Arrhenius behaviour was observed for all samples, with two slopes on two different temperature ranges, 300–400 K and 550–700 K, respectively, which correspond to two activation energies. From the investigation of the complex dielectric constant behaviour (Cole-Cole plots), it was revealed that two relaxation processes are present in the LLTO crystallites, denoted by the authors as b1, respectively, b2 [93]. The first grain relaxation mechanism (b1) is attributed to Li^+^-ion movement from cage to cage. The relaxation frequency of b1 is (for example) 20 MHz at 300 K for the x = 0.12 sample [93]. The second relaxation mechanism is attributed to the rapid Li^+^-ion movement inside the cage. The relaxation frequency (also for example) of b2 is 470 MHz, also at 300 K for the same x = 0.12 sample [93]. The relaxation frequencies for both processes have an Arrhenius type variation with the temperature [93]. The temperature range of the dielectric spectra measurements appears to be 300–400 K.

Interestingly, the activation energies for the relaxation frequencies correlate well with the activation energies of the conductivities. Thus, the activation energy which describes the increase in relaxation frequency with temperature for the b1 process (E_Ab1_) correlates to the activation energy of the grain (bulk) conductivity up to 400 K [93]. Similarly, E_Ab2_ correlates to the activation energy of σ_bulk_ in the high temperature range (550–700 K) [93]. This observation could imply that the process of Li^+^-ion migration in the grain, observed from the impedance spectra at temperatures above 550 K is also observed in the dielectric spectra at lower temperatures (up to 370 K) [93].

Another interesting property of LLTO was evidenced by Bohnké [75], namely the material’s ability to exchange Li^+^ ions with H^+^ ions. The authors observed that LLTO reacts with water and CO_2_ from the atmosphere and forms partly protonated titanates and carbonates at the surface of the sample. The reaction is nevertheless reversible by heat treatment at 300–400 °C. The exchange reaction is faster when the grains size is smaller. In one experiment 2.5 g of LLTO powder containing nanosized grains were dissolved in water and the pH of the solution increased rapidly to 10.3. The increase was much slower, from 5.8 to 8.3 and using 5.5 g of LLTO, when the experiment was performed with powders containing grains in the micrometre range [75]. This is an interesting observation as it highlights a further complication of the system: storage of LLTO in humid atmosphere leads to loss of Li^+^ ions [75].

## 5. Effects of Substitutions

Various substitutions have been attempted on either A site, B site and O site for LLTO from the very early history of the material (1994 [73]) and continuing up to recent times (2022 [36]). The aim of the substitutions is twofold: improving the ionic conductivity of the material and probing the conduction mechanisms in order to gain deeper understanding on the correlation between the material structure and the transport properties.

### 5.1. A Site Substitution

Inaguma not only discovered LLTO, but also tested the effects of various substitutions (for example Ln_1/2_Li_1/2_TiO_3_ where Ln = La, Pr, Nd, Sm), including the La^3+^/Sr^2+^ substitution in the system [(La_1/2_Li_1/2_)_1−x_M_x_]TiO_3_ where M = Sr, Ba as early as 1994 [73]. For the pristine material, it was observed that the conductivity peaks at a Li fraction of 3x = 0.33. It was assumed that conduction occurs through the A-site vacancies. When the Li concentration is increased the number of vacancies decreases, therefore conductivity must present a peak at some point. Inaguma showed, based on the general chemical formula of LLTO, that conductivity is a parabolic function of the Li concentration. The function has a maximum at 3x = 0.25, which is very close to the observed optimum conductivity concentration (3x = 0.33) [73]. The temperature variation of the conductivity was observed to not obey the Arrhenius law, with the activation energy decreasing with the increase in temperature. The effect was explained as the result of the Li ions scattering on the lattice vibrations. At high temperatures the short-range hopping of the Li ions occurs more easily, but the probability of collisions also increases. The central conclusion of the study on the substitution samples is that conductivity is related to the free volume available for the Li ions on the A site. The volume itself depends on the ionic radius of the A-site cation. This is supported by the observations that when the ionic radius of the (non-Li) A-site cation decreases (for the Ln_1/2_Li_1/2_TiO_3_ system) conductivity drops. For the case of the Sr substitution, because the ionic radius of Sr^2+^ ion is larger than the radius of the La^3+^ ion, the lattice constant is increased. The available space for Li movement is also increased. On the other hand, the substitution reduces the Li concentration and therefore conductivity peaks at x = 0.05, where σ = 1.5 × 10^−3^ S/cm [73]. Substitution with Ba^2+^ is found not to increase Li^+^ conductivity despite the larger size of the Ba^2+^ ion. The authors assume that other effects, such as local lattice deformations, compensate the potential gain in conductivity that could result from the addition of the larger ionic radius, Ba^2+^ ion [73].

The La^3+^/Sr^2+^ substitution was also studied by Zhang [6] on a similar LLTO system, namely Li_0.33+x_La_0.56−x_Sr_x_TiO_3_ [6]. The difference between Zhang’s [6] study and Inaguma’s work [73] is that for the former, the Sr substitution increases the Li concentration and in the case of the latter the Li/La ratio was kept fixed, i.e., one Sr ion replaced a Li/La pair and therefore adding more Sr to the system caused a decrease in the Li concentration. For Zheng’s work, the Sr substitution is expected to have a twofold effect: it should increase the lattice size due to the larger Sr^2+^ ionic radius and it should allow an increase in the Li concentration (for charge balance). Samples for x = 0, 0.03, 0.06, 0.09 and 0.12 were prepared and it was observed that the typical LLTO superstructure peak disappears with the Sr doping. The Sr substitution causes the structural transformation of the system from the A-site cation ordered tetragonal system (s.g. P4/mmm) to a disordered cubic system (s.g. Pm3m) [6]. Vegard’s law is obeyed and no Sr containing (or any other) secondary phases are observed in the XRD patterns indicating that Sr ion is fully accepted into the LLTO structure. The observed Nyquist plots were linear for all samples. The effect was attributed to the blocking electrodes and the limited frequency of the EIS instrument (up to 1 MHz). A drop of the activation energy was observed, from E_A_ = 0.35 eV for the undoped samples, to E_A_ = 0.3 eV for the doped samples indicating an enhanced ionic conductivity as the result of the increased available lattice volume. The ionic conductivity was also substantially increased, by a factor of almost 2, between the pristine and the doped samples. For undoped LLTO σ = 9.15 × 10^−4^ S/cm and the highest conductivity in the case of the doped samples reached σ = 1.95 − 1.92 × 10^−3^ S/cm for the x = 0.03, respectively, x = 0.06 samples. The values are obtained at 303 K. Further increase in Sr content leads to reduction in ionic conductivity because it triggers a decrease in the available vacancies. The electrochemical stability of the highest conductivity Sr substitution sample (x = 0.03) was also tested, and it was found that the material remains stable up to 5 V [6].

Recently, Frenandes studied the effects of Eu^3+^/La^3+^ substitution on Li_0.5_La_0.5_TiO_3_ [52]. Because Eu^3+^ can replace La^3+^ on different environments, it is possible to probe the local symmetry of the crystal using photoluminescence spectroscopy [52]. The nature of the chemical bonds within the material (covalent or ionic) was also investigated [52]. The Eu content varied between 0.1 and 1 at%. Li_2_TiO_3_ was observed as a secondary phase for the pristine sample [52]. It appears that Eu promotes distortion and instability. The effect is favourable for sintering, resulting in larger crystallite sizes for lower sintering temperature [52]. No modification in the XRD pattern was noticed between the pristine and the doped samples. Raman peaks associated with Li titanates disappear with the substitution and the observation is explained as the effect of the substitution on the motion of the TiO_6_ octahedra. The study also revealed another possible mechanism for Li-ion transport, meaning that Li^+^ is too small to occupy La^3+^ vacancies and therefore tends to be located at interstitial positions [52]. A decrease in the optical band gap of the substitution samples is observed. This result is the effect of structural disorder induced by the substitution [52]. Concerning the bond characteristics, the study showed that the Li-O bond is more ionic and the La-O bond is more covalent [52].

Abhilash [103] studied the Li/Ag substitution on nanocrystalline LLTO (Li_0.5_La_0.5_TiO_3_). The Ag concentration was varied between 0 and 0.5 with the compositions of the samples defined as follows: Li_0.5_La_0.5_TiO_3_, Ag_0.1_Li_0.4_La_0.5_TiO_3_, Ag_0.3_Li_0.2_La_0.5_TiO_3_ and, finally, complete Li substitution with the Ag_0.5_La_0.5_TiO_3_ sample. In this study, the host material, Li_0.5_La_0.5_TiO_3_, was obtained in the cubic symmetry form with the characteristic superstructure (c = 2 × a). The effect of the Ag substitution is the shift from the cubic superstructure system to a tetragonal system [103]. The (1 0 1) reflection disappears and the intensity of the (1 0 2) reflection, which is specific for tetragonal symmetry, increases with the substitution. Thus, Ag_0.5_La_0.5_TiO_3_ is fully tetragonal while Ag_0.3_Li_0.2_La_0.5_TiO_3_ presents mixed cubic and tetragonal phases [103]. The lattice parameters are a = 3.887 Å for the cubic phase, respectively, a = 4.143 Å and c = 5.432 Å for the tetragonal phase. An increase in the unit cell volume is observed, due to the larger ionic radius of the Ag^+^ ion compared to the ionic radius of Li^+^. Photoluminescence spectroscopy shows no change in electronic structure due to the Ag^+^ substitution. The sample emits blue light (wavelength of 470 nm) when exposed to UV light with the wavelength of 230 nm. The bulk ionic conductivity of the nanopowder is 1.41 × 10^−3^ S/cm and the total conductivity reached 3.094 × 10^−4^ S/cm [103]. When Li is substituted by Ag the conductivity decreases, compared to the LLTO sample without any substitution. The Li ions apparently diffuse faster when Ag is not present. For the Ag_0.1_Li_0.4_La_0.5_TiO_3_ sample, the conductivity already decreased by one order of magnitude compared to the pristine sample. Conductivity continued to decrease for the Ag_0.3_Li_0.2_La_0.5_TiO_3_ sample. The presence of the Ag ions reduces the available vacancies and lowers the occupancy of the Li^+^ ions within the A site of the perovskite cell [103]. Interestingly, the Ag_0.5_La_0.5_TiO_3_ sample, which contains no Li^+^ ions, was found to present the highest conductivity. The authors therefore concluded that a new type of Ag^+^-ion conduction mechanism might be present in that sample [103].

V’yunov and Belous tested the effects of Li/Na substitution in the Li_0.5-x_Na_x_La_0.5_TiO_3_ system (0 ≤ x ≤ 0.5) [69]. The samples were prepared by solid state reaction. For samples where x = 0–0.1 the material shows the coexistence of rhombohedral (space group R-3c) and tetragonal (space group P4/mmm) crystallization systems. Between x = 0.2 and x = 0.5 the system forms a solid solution with rhombohedral symmetry (R-3c space group) [69]. The two crystal systems have the same chemical composition, the difference between them consists in the A-site cation ordering. The tetragonal phase contains cation ordering, but the rhombohedral phase contains randomly distributed A-site cations [69]. No impurity phases are obtained in the final product; however, the material passes through many intermediary phases during synthesis. Ionic conductivity and complex dielectric permittivity were analysed. The main observation is that the Na^+^ ions are fixed and cannot pass through the oxygen bottlenecks—due to the larger Na^+^ ionic radius compared to the Li^+^ ionic radius. Substitution leads to decrease in charge carrier (Li^+^) concentration, decrease in the number of available vacancies and decrease in grain size—which is correlated to an increase in grain boundary resistance [69]. Substitution also causes an increase in the unit cell volume, and for low Na concentrations, the overall effect is increasing the Li-ion conductivity. Further increasing the substitution degree begins to block the conduction pathways and conductivity decreases. The dielectric constant is affected by the Li^+^ transfer mechanisms. The highest attained value was ε ≈ 4 × 10^4^ at 1 Hz for the Li_0.4_Na_0.1_La_0.5_TiO_3_ sample. The competition between the same two processes, increase in Li^+^ ions mobility due to increased bottleneck size and decrease in Li^+^ ions mobility due to the decrease in the number of vacancies, are used to explain the observed non-monotonic dependence of the dielectric constant on the Na content [69].

### 5.2. B-Site Substitution

B-site cation substitutions were also studied. The effects of the Al substitution were studied by Le et al. [1,53]. The two articles were published within less than three years of each other. With both studies, the aim was to improve the grain boundary conductivity of the material (identified as the main limiting factor) by substituting Ti with La and adding excess Li_2_O [1,53]. Li_2_O is used a flux to improve crystallization. The composition of the material studied for both works was (Li_0.33_La_0.56_)_1.005_□_0.106_Ti_0.99_Al_0.01_O_3_ [1,53]. The samples were prepared by the citrate-gel method. No impurity phases were observed [1,53]. The two studied focused on slightly different aspects. The first article explores the material properties and the behaviour of the material in a symmetrical cell [53]. The second article explores the possibility of using the material as an electrolyte for an aqueous rechargeable lithium metal battery [1]. Parallels are drawn, but the results are mostly discussed separately.

For relatively short sintering times (6 h) the sample is tetragonal with the usual space group P4/mmm and lattice parameters a = 3.874 Å and c = 7.746 Å, perfectly matching the Li_0.33_La_0.56_TiO_3_ peaks [53]. It is, however, noted that lattice parameters (and cell volume) present a monotonous increase with the Li_2_O content [53]. For example, the lattice constant “a” varies between a = 3.873(2) Å for no Li_2_O excess to a = 3.877(2) Å for 35 mol % excess Li_2_O. It was observed that the grain size increases for more than 15% Li_2_O added [53]. The ionic grain conductivity for the un-doped sample was determined to be 8.48 × 10^−4^ S/cm and the grain boundary ionic conductivity was 1.06 × 10^−4^ S/cm [53]. The authors report a peak of conductivity at 20% excess Li_2_O with σ_bulk_ = 2.99 × 10^−3^ S/cm and σ_grain boundary_ = 3.55 × 10^−4^ S/cm [53]. At the same amount of Li_2_O the activation energy presents a minimum with E_A-bulk_ = 0.214 eV and E_A grain boundary_ = 0.382 eV. Excess Li_2_O increases and then decreases conductivity. With the addition of Li_2_O, the grain size increases, and the grain boundary area decreases (which favours ionic conductivity); however, too much Li_2_O leads to the formation of pores and this blocks the Li^+^ motion, lowering conductivity. Moreover, Li_2_O increases Li^+^ concentration but lowers the vacancies concentration [53].

Increasing sintering time (12 h) leads to the formation of the cubic system with the characteristic superstructure [1]. Le et al. continued to examine the effects of sintering time on the conductive properties as well [1]. They observed that increasing the sintering time increases conductivity by reduction in the resistive grain boundaries [1]. A similar effect is obtained by increasing the sintering temperature [1]. The highest conductivity values are obtained at 6 h sintering time: σ_total_ = 3.17 × 10^−4^ S/cm, σ_grain boundary_ = 3.35 × 10^−4^ S/cm and σ_bulk_ = 2.99 × 10^−3^ S/cm [1]. The Arrhenius plot is linear for all samples and the lowest activation energy is observed also at 6 h sintering time with E_A_ = 0.358 eV [1]. Very interestingly, the time dependence is not linear. Higher sintering times reduce conductivity. Longer times favour crystallite growth; however, Li evaporation is significant and vacancies become too abundant. Li evaporation is confirmed by ICP [1]. For this reason, the best conductivity is achieved at 6 h sintering time, even though the crystallites are not the largest under these conditions. Moreover, the density decreases with the sintering time leading to more defects in the crystal structure and higher porosity—hampered Li conduction and higher activation energies [1]. Another highly interesting aspect emphasised by Le [1] is the H^+^/Li^+^ topotactic exchange on the perovskite structure. The authors noticed severe degradation of conductivity for samples immersed in neutral and slightly acidic environments [1].

There is another extremely important aspect concerning the possibility of using LLTO as an electrolyte within a lithium-ion battery. LLTO intercalates Li during the functioning of the battery, and this leads to the reduction of Ti^4+^ to Ti^3+^. The reduction increases the electronic conductivity of LLTO, and this can eventually lead to the formation of an internal short-circuit. One possible solution to this problem is to replace Ti^4+^ with Nb^5+^, and thus obtain a compound with the composition Li_x_La_(1−x/)/3_NbO_3_ (LLNbO). Hu et al. [104] explored the effect of annealing temperature on the structural and ionic conductivity properties of LLNbO single crystals. The characterization of the structure of LLNbO is beyond the scope of this review article; however, some details have to be explained. The structure of LLNbO is different from that of LLTO in the stacking of the A-site ions. For LLNbO two crystallographic layers are observed, the first layer (A1) is partly occupied and contains the A-site ions and vacancies (the occupancy is 2/3 La + 1/3 vacancy) and the second layer (A2) is completely vacant [104]. The authors worked on single crystals [104]. Because on a single crystal grain boundaries are eliminated, the conductivity of LLNbO can be enhanced [100,105,106] up to 2.2 × 10^−4^ S/cm [104]. Moreover, Nb^5+^ replaces Ti^4+^, and this leads to an increase in the number of A-site vacancies. Higher Li conductivity is expected, but is not observed [104]. The lack of conductivity is explained as the result of the confinement of Li ions to the La occupied layer. Thus, LLNbO becomes a quasi-2D ionic conductor with conduction occurring only through the A1 layer. This is in contrast to LLTO where the conduction occurs in both A1 and A2 layers [104]. It was also observed that all structural changes are detrimental to Li^+^ conduction, and that annealing could induce such changes. After annealing the distribution of A-site ions and vacancies in the A1 layer becomes less well defined [104] and the size of the nanodomains decreases [104]. As a result, ionic conductivity decreases by 15% after annealing [104].

### 5.3. Oxygen Site Substitution and Other Substitutions

Substitutions were also studied on the oxygen site, namely O^2−^/F^−^ substitutions. The review article will quickly discuss two examples of this substitution type.

Li [74] studied the effects of the anion substitution in the Li_3x−y_La_2/3−x_TiO_3−y_F_y_ (x = 0.11, 0 ≤ y ≤ 0.183) system as early as 2005. The samples were prepared by solid state reaction. The main phase was identified as the tetragonal phase (space group P4/mmm, a = a_p_, c = 2a_p_ where a_p_ is the lattice constant of the primitive perovskite unit cell) with the La-rich/La-poor superstructure along the c axis. The lattice parameters vary monotonously with the F^−^ concentration (between a = 3.871 Å, c = 7.749 Å for y = 0 and a = 3.869 Å, c = 7.756 Å for y = 0.183) [74]. Small amounts of monoclinic Li_2_TiO_3_ are observed as secondary reaction products. High (up to 40%) LiF evaporation was observed. However, it could be inferred that F is accepted into the LLTO structure [74]. The ionic conductivity is improved with a maximum from σ_bulk_ = 1.01 × 10^−3^ and E_A_ = 0.366 eV for y = 0 at T = 298 K to σ_bulk_ = 1.59 × 10^−3^ S/cm and E_A_ = 0.346 eV for y = 0.072 at the same temperature. Further increase in F concentration decreases conductivity to σ_bulk_ = 4.91 × 10^−4^ S/cm and the activation energy rises again to E_A_ = 0.369 for y = 0.183 [74]. Above 400 K the conduction mechanism changes from the thermally activated Arrhenius process to the thermally assisted Vogel–Fulcher–Tammann process [74]. The explanation for the variations in conductivity with the F^−^ intake is based on the change in bond strength due to the anion substitution. The Ti-O bond distance decreases with the substitution (the bond strength increases), and this lowers the strength of the Li-O bond, and hence increases the conductivity. Further increase in F results in the decrease in the product between charge carriers and vacancy concentration [74].

Okumura increased the Li^+^-ion conductivity in LLTO by inducing A-site disorder through addition of LiF during synthesis [5]. The authors studied two sets of samples with the general compositions of Li_0.33+3y_La_0.56−y_TiO_3_ and Li_0.33_La_0.56−y_TiO_3−3y_F_3y_ [5]. No impurities were observed. The samples crystallized under the cubic symmetry, space group Pm3m. The diffraction peaks characteristic for the superstructure were not observed, thus indicating a random distribution of Li, La and vacancies. The lattice parameter is constant with y for the pristine samples and slightly decrease with y for the substitution samples [5]. The ionic conductivity decreases with y for the pristine samples; however, in the case of the substitution samples the ionic conductivity increases with y. The peak conductivity is achieved for y = 0.017 and reaches 2.30 × 10^−3^ S/cm at 30 °C [5]. X-ray absorption near edge structure (XANES) analysis showed that Ti^4+^ is not reduced by the presence of F, and thus the electronic conductivity of the material, formed by the Ti^3+^-O^2−^ framework, is expected to be negligible [5]. Some explanations are proposed for the improving of ionic conductivity, these include site percolation effects, differences of local environment around the Li ions and the possible formation of amorphous compounds caused by the addition of the Li salt [5].

LLTO itself can be used as a dopant. Hua et al. [36] studied the effects of doping with LLTO (Li_0.5_La_0.5_TiO_3_) on the sintering characteristics, phase structure, optical properties and electrical properties of the following complex material: (1 − x)(0.94K_0.51_Na_0.5_NbO_3_ − 0.06SrZrO_3_) − xLi_0.5_La_0.5_TiO_3_. The base material is (K_0.51_Na_0.5_)NbO_3_ (KNN). The KNN materials are used in photoelectric devices because of their high transparency and photosensitive resistance. The samples obtained by Hua were synthesised by solid state reaction. No secondary products were detected. The authors note the effects of the reactants, thus: La_2_O_3_ reduces porosity and defects and yields samples with excellent electrical and optical properties, Li_2_O (similarly to the example above [1,53]) is a sintering aid used with the role of reducing the sintering temperature. LLTO reduces sintering temperature, increases density and widens the sintering temperature range [36]. Concerning the optical properties, it was observed that the highest transmittance is obtained at x = 0.02 for all wavelengths above 350 nm. Higher or lower LLTO content decreases transmittance. The observation is explained as the effects of sample density and grain size in that porosity increases scattering [36]. It was also observed that the impedance of the sample when the sample is illuminated is significantly lower than the impedance of the sample without exposure to the light source, i.e., electrical conductivity increases with the incident light [36].

## 6. Composite Electrolyte

**Figure 11:**
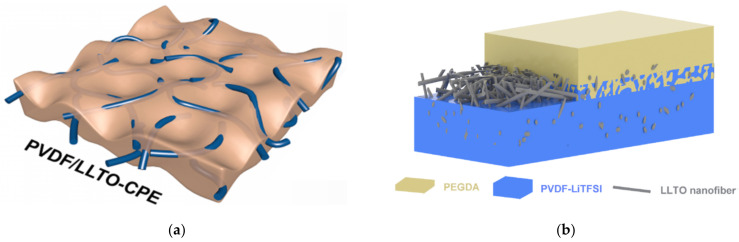
(**a**) Representation of composite electrolytes consisting of LLTI nanofibers dispersed in a polymer matrix from references [107], (**b**) Representation of composite electrolytes consisting of LLTI nanofibers dispersed in a polymer matrix from references [4]. Reprinted with permission. Copyright 2021 Applied Surface Science—Elsevier and, respectively, 2022 Journal of Power Sources—Elsevier.

**Figure 12:**
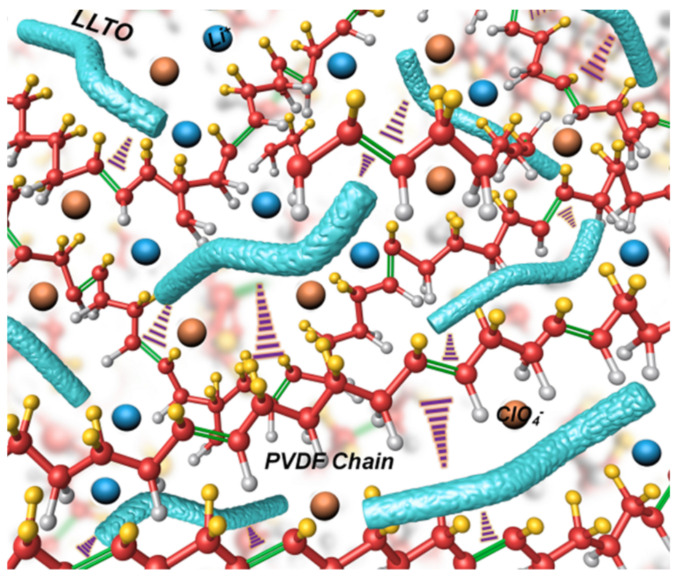
Atomic scale representation of a composite electrolyte from reference [107]. Reprinted with permission. Copyright 2021 Applied Surface Science—Elsevier.

It has been shown during the previous sections that according to the research, currently, the main problem impeding the implementation on a large scale of solid-state electrolytes (particularly LLTO) for Li-metal batteries is not the intrinsic ionic conductivity of the electrolyte, but rather the loss of conductivity at the interface between grains. Polymer electrolytes have their own limitations: low mechanical strength, poor conductivity and lower cycle stability due to structural change in the polymer chains [107]. Recent research (roughly 2018–2022) has focused on the development of organic–inorganic composite materials. The ionic conductivity mechanisms are different between organic (polymer) and inorganic (ceramic) compounds. In a composite electrolyte the two types of materials should compensate for each other’s limitations. With an over-simplification of the problem, it has been shown (for the case of LLTO) that conduction occurs through the four-oxygen-ion bottlenecks, and it stops at the crystallite boundaries, while the conduction in a polymer occurs through the motion of the polymer chains (but also along the chain). If crystallization of the polymer begins to form, the motion of the polymer chains is restricted and conductivity decreases [108]. Figure 11 shows representations of the microscopic structure of composite electrolytes from references [4,107]. An atomic scale representation is provided in Figure 12 according to reference [107]. It has been observed [9,10,11] that the addition of some ceramic particles to the polymer matrix limits the ability of the polymer to crystallize and therefore improves conductivity. In a composite material (obtained by mixing a fast ionic conductor ceramic and a conductive polymer), the polymer could bridge the gap between the ceramic particles, and thus ease the ion transfer and at the same time the ceramic particles could reduce the crystallization tendency of the polymer, therefore improving conductivity. Additionally, the polymer could improve the electrode–electrolyte contact interface and prevent the oxidation/reduction in the ceramic electrolyte at the contact with either electrode [3]. Ti from LLTO is known to be rapidly reduced from Ti^4+^ to Ti^3+^ when LLTO is in contact with metallic lithium [61] due to the Li ions occupying the available vacancies [61]. This increases the electronic conductivity of the material [61]. As it will be shown in the next examples, there is a limit to the increase in conductivity achievable through mixing conductive ceramics and polymers, and peak conductivity is observed for a certain polymer/ceramic ratio. Generally, the polymer conductivity is lower than the ceramic conductivity so if the composite contains too much organic material the conductivity will decrease. On the other hand, if the composite contains too much ceramic material, agglomeration of the ceramic particles occurs, and conductivity will decrease again. 

Throughout the examples, this review will focus mainly on the values of Li-ion conductivity with various electrolyte properties, but also the Li-ion transference number will be investigated. The transference number shows the fraction of the total electric current carried by the Li ions. It is obtained using an expression of the following form—Equation (5):

(5) $$t_{{Li}^{+}}=\frac{I_{S}\left(\Delta V-I_{0}R_{0}\right)}{I_{0}\left(\Delta V-I_{S}R_{S}\right)}$$

where I_S_ represents the steady state current, I_0_ represents the initial current, ∆V is the applied voltage, R_S_ is the polarised resistance and R_0_ is the resistance before polarization.

### 6.1. LLNO Nanowires—Polymer Composite Electrolytes

Nourisabet [109] synthesised and tested a polyethylene oxide–polyvinylidene fluoride (PEO-PVDF) blend to which LLTO (Li_0.35_La_0.55_TiO_3_) nanowires were added. The nanowires were obtained by electrospinning and had diameters of approximately 88 nm, 161 nm and 238 nm. Blending the polymers and adding the ceramic filler are known techniques for reducing the concentration of the polymer crystalline phase [109,110,111]—thus improving not only conductivity but also mechanical strength [109]. The authors determined the polymer blend composition that generates the highest conductivity (a mixture containing 6 wt% PEO and 6 wt% PVDF) and added LLTO nanowires to that specific polymer blend [109]. The ionic conductivity is observed to increase with the addition of the LLTO nanowires. The increase depends on the diameter of the nanowire and is 12, 8 and, respectively, four-times higher than the conductivity of the best polymer blend. The conductivity is inversely proportional to the wire diameter. Conductivity is 12-times higher for PEO-PVDF-LLTO than for PEO-PVDF when the nanowires diameter is 88 nm (the other increases are respective to the wire diameters). The peak conductivity is 6.02 × 10^−3^ S/cm for 88 nm wires and the transference number is 0.861. Maximum conductivity is achieved when the nanowire weight concentration reaches 8%. Any more LLTO added to the matrix leads to agglomeration. Less LLTO added results in fewer channels [109]. Nyquist diagrams measured for the system are linear at high frequency and this indicates that the conduction is ionic [109]. Some of the mechanisms that could lead to the increase in conductivity include: increase in Li transference number due to Li-bond in the ceramic structure, the quasi 1D nature of the nanowires could increase the number of mobile Li ions, additional ion transport pathways through LLTO, the ability of LLTO to absorb anion species that otherwise reduce the electrolyte stability window and the ability of LLTO to alter the polymer structure at the polymer/nanowire interface [108,109,112,113,114].

Li [107] experimented with a PVDF/LLTO system inspired by the dragonfly wing structure. The article contains both the theoretical approach and the experimental investigation. For the experimental part, the structure was obtained by embedding 1D LLTO nanowires (obtained by electrospinning) in a PVDF membrane [107]. This configuration achieved great mechanical strength of 10 MPa and good contact to the metallic Li anode [107]. Batteries containing the PVDF/LLTO composite polymer electrolyte (CPE) were constructed and showed good cycling stability with capacity of 140 mAh/g after 200 cycles at a rate of 0.2 C [107]. The activation energy was measured to E_A_ = 0.264 eV [107]. The ionic conductivity measured as a function of the LLTO concentration shows a peak value of σ = 5.8 × 10^−4^ S/cm at 15 wt% LLTO filler [107]. The membrane was designed to have a thickness of 25 µm. The low thickness could reduce the Li^+^ transport path, and thus improve conductivity [107]. Other reasons for the improved conductivity include induced partial dehydrofluorination of PVDF (by the introduction of LLTO), which enhances the interaction between LLTO and PVDF and reduces the crystallinity of the PVDF matrix. The electrolyte also contained LiClO_4_. It is possible that the presence of the LLTO nanowires and the dehydrofluorinated PVDF could favour the dissociation of LiClO_4_ and therefore cause and increase in the Li^+^ charge carrier concentration [107]. It was also observed that when the LLTO concentration is increased (up to 25%) the ionic conductivity is decreased. The observation is explained as the effect of agglomeration [107].

Another (earlier) experiment on the properties of the PEO/LLTO-nanowires composite was carried out by Zhu [108]. The authors prepared an electrolyte containing PEO, LiTFSI and LLTO (Li_0.33_La_0.557_TiO_3_) nanowires obtained by electrospinning and calcination. The entire electrolyte was obtained by the solution casting method [108]. The LLTO phase was identified by XRD. No peak shift was observed as a result of nanowire inclusion. However, peak intensity was observed to decrease for both PEO and LLTO on the PEO-LiTFSI-(5%) LLTO sample. The addition of LLTO lowered the glass transition temperature (T_g_ = −45.8 °C) and lowered the melting temperature (T_m_ = 54.5 °C) for the CPE up to 5 weight % addition of LLTO. At higher LLTO concentrations T_g_, T_m_ and crystallinity began to rise again [108]. The 5% LLTO addition is the optimum concentration for conductivity. The conductivity also rises with the temperature. For the sample containing 5% LLTO nanowires added, the conductivities are as follows: σ = 5.53 × 10^−5^ S/cm at 25 °C and σ = 4.65 × 10^−4^ S/cm at 60 °C. By contrast, for pure PEO, the conductivity is σ = 1.06 × 10^−5^ at 25 °C and σ = 3.17 × 10^−4^ S/cm at 60 °C [108]. Small amounts of LLTO did not increase much the conductivity. Larger amounts of LLTO decreased conductivity. It was observed that accumulation of LLTO nanowires is not conductive for Li^+^ ions. The activation energy has different values above and below 50 °C which is the recrystallization temperature for PEO [108]. Possible mechanisms suggested by Yi in order to explain the increase in ionic conductivity could be: quasi 1D characteristic of LLTO wires increasing the content of mobile Li^+^ ions, LLTO adds paths for Li^+^-ion migration, LLTO attracts anions and prevents oxidation of LiTFSI, LLTO could change the structure of the polymer chain at the interface [108]. The authors also mention further effects of the LLTO addition to the stability of the system. TGA analysis showed an increase in the electrolyte decomposition temperature (from 460 °C for PEO to 500 °C when LLTO is added, LLTO nanowires themselves remain stable below 600 °C). The increase in the decomposition temperature is probably due to increased specific heat of the system caused by the presence of LLTO [108]. The electrochemical stability of the CPE increased by at least 4.74 V when LLTO nanowires are added [108].

Yang [115] prepared Li_0.33_La_0.56_TiO_3_ nanofibers with a diameter of approximatively 108 nm by electrospinning and calcination at 900 °C. The nanowires were then embedded in polyvinylpyrrolidone (PVP). Oxygen vacancies were introduced into the LLTO perovskite nanofibers by hydrogen treatment. The ionic conductivity was then measured as a function of the hydrogen treatment. The conductivity was observed to rise with increasing temperature of the treatment as follows: σ = 2.6 × 10^−4^ S/cm for the pristine sample, σ = 3.3 × 10^−4^ S/cm for the H-LLTO sample obtained at 400 °C, σ = 4.8 × 10^−4^ S/cm for the H-LLTO sample obtained by treatment at 500 °C and σ = 4.9 × 10^−4^ S/cm for the H-LLTO sample prepared at 600 °C [115].

Zhu [116] studied another polymer-ceramic CPE, namely polyvinylidene flouride-cohexafluoopropylene/polypropylene carbonate—Li_0.35_La_0.557_TiO_3_ nanorods composite electrolyte (PVDF-HFP/PPC/LLTO). The LLTO nanorods are obtained by electrospinning. The authors tested the system for different weight rations of LLTO nanorods (5%, 8%, 10% and 13%) [116]. The ionic conductivity of the sample reaches a maximum of 2.18 × 10^−4^ S/cm at a concentration of LLTO of 10 wt%. Again, it is observed that at higher concentrations of LLTO ionic conductivity begins to decrease. The decrease can be attributed to the agglomeration of nanorods which hinders ion migration [116]. The authors also tested symmetrical Li/CPE/Li batteries and observed that the material presents a high ion migration number of 0.47 and that the battery has excellent cycling stability for 2000 h at 0.1 mA/cm^2^ [116].

An earlier example of LLTO obtained by electrospinning is seen in the works of Yang [56]. The authors prepared LLTO nanowires with a diameter of 100—200 nm by calcination at 1000 °C for 3 h. The LLTO sample obtained through this process crystallised in the tetragonal structure with the P4/mmm space group, and the lattice constants: a = 3.875 Å and c = 7.739 Å. The bulk ionic conductivity was determined to be in the order of 10^−4^ S/cm, which is comparable to values measured for LLTO samples obtained by solid-state reaction. Another interesting aspect noticed by the authors is that LLTO nanopowders, with grains of 15–20 nm diameter, prepared using a combustion method, showed an extremely low grain boundary conductivity in the order of 10^−10^ S/cm at room temperature [56].

### 6.2. Oxid—Oxide Hybrid Electrolytes

Fully inorganic, oxide–oxide composites were also studied. For example, the works of Song [117] focused on the study of the LAGP-LLTO composite, where LAGP is Na super ionic conductor (NASICON) with the composition Li_1.5_Al_0.5_Ge_1.5_(PO_4_)_3_, and here it serves as the matrix of the system (and LLTO is the additive) [117]. The two compounds can react with each other. The reaction is based on the Ti^4+^/Ge^4+^ substitution. The Ti^4+^ ionic radius is larger than the ionic radius of Ge^4+^ and as a result the substitution causes shifting of the diffraction peaks. The two materials (LAGP and LLTO) form a solid solution with the general equation Li_1+x_Al_x_Ge_2−x−y_Ti_y_(PO_4_)_3_ [117]. The maximum ionic conductivity of the composite is σ = 4.04 × 10^−4^ S/cm for an LLTO concentration of 4 wt%. The explanation formulated by the authors for the improved ionic conductivity is related to the decomposition of LLTO and generation of LaPO_4_. These effects lead to the formation of a space charge layer at the matrix/LaPO_4_ interface and the presence of the space charge layer leads to the improvement of conductivity [117].

Another example of oxide–oxide hybrids can be found in the works of Yi [83]. Yi et al. studied the properties of LiNi_0.5_Co_0.3_Mn_0.2_O_2_ microscopic spheres coated with LLTO (Li_0.5_La_0.5_TiO_3_) [118]. The coating is amorphous and has a thickness in the order of 20 nm. The base material is a battery cathode. The LLTO coating has the role of improving the cathode/electrolyte interface. The improvement consists of: accelerated Li^+^ migration rate at the cathode, suppression of the space charge layer formation and inhibited decomposition of the electrolyte [118].

### 6.3. Dendrite Growth Suppression and Chemical Stability

Liu [119] noticed that high conductivity and high stability tend to be mutually exclusive and tested the trade-off between these two properties on the vermiculite-Li_0.33_La_0.557_TiO_3_-PEO (Vr-LLTO/PEO) composite material [119]. Vermiculite was obtained as nanosheets that were later uniformly dispersed in a gel solution containing precursors of LLTO. The resulting Vr-LLTO structure was then loaded by dripping and drying with a PEO/LiTFSI solution until the cavities of Vr-LLTO were completely filled with the PEO/LiTFSI solution [119]. XRD confirmed the embedding of LLTO into Vr and of Vr-LLTO into PEO. No structural change was observed to either Vr or LLTO [119]. Conductivity was determined for pristine LLTO at σ = 4.79 × 10^−5^ S/cm and for the Vr-LLTO/PEO CPE at σ = 1.04 × 10^−4^ S/cm at a temperature of 25 °C [119]. LLTO adheres to the Vr nanosheets and accordingly forms long continuous Li^+^-ion transfer paths, consequently reducing the grain boundary resistivity and therefore the ionic conductivity and the Li transference number increase significantly [119]. Another effect of the CPE’s structure is that PEO prevents reduction of Ti^4+^ to Ti^3+^ (as evidenced by XPS investigations), and this improves the stability of the interface between the Vr-LLTO/PEO CPE and the metallic Li anode. In the absence of the PEO layer, LLTO undergoes reduction at the contact with metallic Li [103]. We will mention here only one more observation from the same work, namely the fact that the mechanical properties of the CPE (tested by nanoindentation) are also improved and the Vr-LLTO/PEO system shows great resistance to dendrite growth [119].

Li et al. [2] tested LLTO/PVDF heterostructures consisting of stacked 75 wt% LLTO/PVDF and, respectively, 15 wt% LLTO/PVDF. The composition of LLTO was Li_0.35_La_0.55_TiO_3_. Concentrations of 75% (for LLTO-75) and, respectively, 15% (for LLTO-15) refer to the concentration of LLTO in the LLTO/PVDF CPE. The heterostructure is formed from one LLTO-75 layer sandwiched between two LLTO-15 layers. LLTO-75 presents high mechanical strength, resistance to dendrite growth and high ionic conductivity. It consists mostly of an LLTO network with PVDF embedded through the matrix. LLTO-15 is a softer, more flexible and electrochemically stable composite that forms a good contact interface with the electrodes [2]. The LLTO nanofibers have good crystallinity as confirmed by XRD and SAED. The ceramic material was obtained in the cubic system. The crystallinity of the polymer was reduced by the addition of the LLTO filler [2]. The ionic conductivity was tested for four samples, namely pristine LLTO, LLTO-15, LLTO-45 (45 wt% LLTO in LLTO/PVDF) and LLTO-75. The conductivities were measured as follows (at 25 °C): σ = 4.2 × 10^−5^ S/cm for LLTO, σ = 5.25 × 10^−4^ S/cm for LLTO-15, σ = 7.8 × 10^−5^ S/cm for LLTO-45 and σ = 8.1 × 10^−5^ S/cm for LLTO-75. The peak conductivity is clearly observed for LLTO-15 (higher by one order of magnitude). Increase in LLTO above 15 wt% causes a decrease in miscibility with PVDF and hence lowering of conductivity [2]. The battery constructed around the LLTO/PVDF heterostructure exhibits a specific capacity of 108 mAh/g at 1C and 91.7% capacity retention after 1000 cycles [2].

LLTO-based CPEs can also be utilised for the construction of Li-S batteries as shown by the works of Kou [120]. Kou et al. studied the LLTO-PEO/LiTFSI CPE from this context. They highlighted the limitations of hybrid electrolytes particularly in terms of mechanical strength/resistance to dendrite growth. They fabricated an asymmetric LLTO framework consisting of porous layers and dense layers. The dense layer contains higher Li concentration and is responsible for facilitating the Li^+^ uniform deposition and therefore suppressing dendrite growth [120]. It also functions as a physical barrier for dendrite growth and considerably enhances the compression strength of the CPE structure. The Li-rich, dense phase contains 72.5 wt% LLTO and the Li-poor, porous phase contains 10 wt% LLTO. The LLTO composition is Li_0.35_La_0.55_TiO_3_. SEM imaging shows that the dense phase achieves low grain boundary resistance due to the close packing of the grains [120]. XRD showed that the obtained LLTO phase is crystallised in the tetragonal system. As in the other cases, PEO crystallinity is reduced by the presence of the LLTO framework. Then, LLTO is also responsible for adding conduction pathways for the Li^+^ ions. The obtained ionic conductivity was 1.49 × 10^−4^ S/cm at 30 °C, and the transference number reached 0.57 [120]. Moreover, in agreement with other studies, at LLTO concentrations above 10 wt%, conductivity begins to decrease [120].

Zhao [121] prepared a CPE based on Li_0.33_La_0.557_TiO_3_ nanofibers obtained by electrospinning embedded on a polyethylene carbonate (PEC) matrix. LLTO presented a cubic structure with homogeneous morphology when calcinated at 800 °C. The ionic conductivity was highest (σ = 3.48 × 10^−1^ S/cm) for a LLTO concentration of 5 wt% when the nanowire diameter is 250 nm [121]. The authors also observed that the tensile strength and the elongation at breaking of the CPE are reduced by 10%, respectively, 20% compared to the corresponding values obtained for the pure PEC electrolyte [121].

Mechanically blocking the dendrite growth is good strategy, but other alternatives are also imagined. For example, the electrolyte chemistry could favour the dissolution of the dendrites and the uniform deposition of Li, thus enabling so called self-healing characteristics of the electrolyte. LLTO-based CPE could be used within this context as proven by Li et al. [3]. Li prepared a Li_0.35_La_0.55_TiO_3_/PEO electrolyte to which fluoroethylene carbonate (FEC) was added. The FEC assists the self-healing process. The over-simplified self-healing mechanism is based on the tendency of FEC to be driven to the nucleation point of dendrite growth. At the nucleation site FEC decomposes and forms a new and even LiF-rich layer which prevents the growth of the dendrites [3]. The electrolyte is obtained by producing an LLTO framework. PEO is dissolved in acetonitrile and dripped onto the LLTO framework (the solvent then evaporates) [3]. The LLTO phase is identified both by XRD and TEM. No impurities are found. However, it is noted by the authors that two calcination steps are required for obtaining a 3D Li conducting LLTO structure. A single calcination step only produces many 2D, sheet, structures which break continuity and lower ionic conductivity [3]. The material presents both ionic conductivity and electronic conductivity. The electronic conductivity is low, but it rises sharply with the temperature. The optimum temperature is between 25 and 50 °C where the ionic conductivity is high, and the electronic conductivity is low. At 25 °C the values for the two types of conductivities are σ_ionic_ = 1.13 × 10^−4^ S/cm (even higher at 50 °C) and, respectively, σ_electronic_ = 1.68 × 10^−9^ S/cm [3]. The authors also noted that impedance changes with the number of cycles [3].

Xu et al. [122] also tested a new mechanism for dendrite suppression. Here the suppression is achieved through both mechanical barriers and in-situ chemical suppression [122]. The CPE designed by Xu and collaborators consist of a 3D fluorinated perovskite-type electrolyte hybridized with polyethylene oxide fibres (F-LLTO-PEO). The base material here is tetragonal LLTO (s.g. P4/mmm) with F^−^/O^2−^ substitution. Theoretical approach (DFT) as well as experimental results (XRD, NPD, TEM, SAED) confirm the inclusion (successful substitution) of F^−^ into the structure [122]. It was calculated by DFT that the composite material should present a higher band gap, and hence wider electrochemical stability window, than pristine LLTO (E_gap_ = 2.30 eV for F-LLTO and E_gap_ = 2.26 eV for LLTO). The fluorinated sample also presents better ionic conductivity (σ = 5 × 10^−4^ S/cm for F-LLTO) than the pristine counterpart (σ = 1.2 × 10^−4^ S/cm for LLTO) [122]. Stability is improved as well with F-LLTO stable up to 6 V, contrary to LLTO, which begins to decompose at 4.8 V [122]. Batteries constructed based on the F-LLTO-PEO electrolyte present exceptional characteristics, such as: high-rate capability of 1 mA/cm^2^, interface stability towards Li metallic anodes > 1000 h under 0.1 mA/cm^2^, rate capacity of 95 mAh/g at 5C and cycling capacity retention of over 80% after 100 cycles at 90 °C [122].

Liu et al. [4] studied yet another possible configuration for a LLTO-based CPE, with applications to high voltage solid lithium-metal batteries. The base material in their study remains Li_0.35_La_0.55_TiO_3_ produced in the form of a nanofiber framework coated on both sides with different polymer electrolytes in order to meet mutually exclusive electrochemical stability requirements, namely, the electrolyte must be reduction-resistant at the anode interface and oxidation-resistant at the cathode interface. Thus, one side contains reduction tolerant polyethylene glycol diacrylate (PEGDA) and the opposite side contains oxidation resistant polyvinylidene fluoride (PVDF) [4]. LLTO provides both high ionic conductivity and mechanical strength. It is interesting to observe that no distinct interface is formed between the two polymers due to their reciprocal solubility [4]. Moreover, it should be noted that small amounts of LiTFSI are added on both sides. The activation energy for the Li-ion transport through this electrolyte is E_A_ = 0.23 eV. The double layer electrolyte presents wide electrochemical stability window of 0–4.5 V with reference to a Li/Li^+^ electrode (unlike typically solid polymer electrolytes, which have a narrow electrochemical stability window). The ionic conductivity of the material is 0.1 × 10^−3^ S/cm at room temperature [4]. Some impedance fluctuations with cycling were observed, similar to the case of liquid electrolytes. There is an initial increase in impedance due to the degradation of the liquid electrolyte followed by the formation of a SEI layer. At later stages, impedance decreases again due to the improved contact at the electrode interface through repeated stripping and plating [4].

### 6.4. Organic Matrix—LLTO Composites

Feng and Lin studied the properties of a polyethylene separator coated with LLTO (Li_0.35_La_0.55_TiO_3_) [123]. The XRD pattern of the material did not reveal the presence of any impurities. All the observed peaks were indexed with the cubic phase of LLTO; however, the superstructure peaks are visible [123]. The ionic conductivity of the PE-LLTO CPE was 0.38 × 10^−3^ S/cm [123]. The high conductivity value is caused by the larger electrolyte uptake, and possibly due to the presence of LLTO [123]. The batteries assembled by the authors, based on this CPE, have excellent cycle stability with 88.7% capacity retention after 1000 cycles [123].

A very interesting potential use for LLTO, is evidenced by Zhao [37]. Here LLTO refers to Li_0.5_La_0.5_TiO_3_. The ceramic is used with the role of semiconductive shielding in HVDC cables where it helps to dissipate the space charge that otherwise accumulates on the insulation layer. The authors obtained cubic LLTO with the superstructure, as confirmed by XRD. Almost no impurities are observed in the final product. The intensity of diffraction peaks corresponding to impurity phases decreases with increasing temperature. Moreover, crystallinity improves [37]. The best space charge reduction is achieved at a LLTO concentration of 4 wt% [37]. The space charge reduction effect is explained as the result of Li^+^-ion migration which forms centres where electrons are absorbed by Coulomb interaction [37]. The tensile strength of the insulation remains good after LLTO insertion in the matrix [37].

## 7. Thin Films and Lamellar Structures

Considerable effort has been invested in the study of LLTO thin films. This topic is not the scope of our review, but some of the work on the study of thin films will be exemplified below.

Lv [33] prepared LLTO-In_2_O_3_ nanorods by electrospinning for the fabrication of new LLTO-based H_2_S gas sensors. The pure LLTO obtained has the composition Li_0.5_La_0.5_TiO_3_, and it was crystallised in the cubic system. The material retained the cubic structure within the LLTO-In_2_O_3_ composite. In_2_O_3_ itself is also cubic. TEM confirmed the formation of both phases, with the lattice spacing of d = 2.92 Å corresponding to the In_2_O_3_ (2 2 2) planes, respectively, d = 2.75 Å corresponding to the LLTO (1 1 0) crystallographic plane [33]. The sensing mechanism is based on the change in resistivity due to the concentration of oxygen. The oxygen vacancy (O_V_) concentration is significantly higher in LLTO-In_2_O_3_ than in pure LLTO. Therefore, the active sites on the appearance of LLTO-In_2_O_3_ are increased, which accelerates the chemisorption of O^2−^ and the generation of oxygen responsible for conduction, chemisorbed oxygen (O_C_) (O^−^ and O^2−^). During the sensing process the number of electrons recombined with O_C_ on the surface of LLTO-In_2_O_3_ is significantly increased, and this increases the LLTO-In_2_O_3_ resistance, therefore improving the H_2_S sensing performance (over pristine LLTO) [33].

Another very interesting approach is obtaining LLTO lamellar structures using a metal–organic-framework (MOF). The works of Dong [124] are a good example of a study of this technique. The authors obtained MOF lamellar membrane derived LLTO electrolyte by embedding LLTO precursors into the pores and interlayer channels of the MOF membrane, followed by in situ sintering [124]. TiO_2_ peaks appeared on the XRD patterns of the MLLTO structure. The interplanar spacing for the structure is d = 7.8 Å for MOF and d = 8.8 Å for MLLTO (indicating that LLTO is successfully embedded in the MOF structure) [124]. MLLTO achieves high ionic conductivity of 1.19 × 10^−4^ S/cm at RT which is much higher than the conductivity of conventional electrolytes. The Li/MLLTO/Li symmetrical cell can stably cycle for 1000 h at current density in the range of 0.1–0.4 mA/cm^2^ at 60 °C without obvious polarization [124].

Xiong [78] obtained LLTO thin films deposited on ITO/glass substrate by RF sputtering. The film composition is Li_0.33_La_0.57_TiO_3_. The films were annealed at 400 °C. The ionic conductivity of the LLTO thin film increases with the annealing operation from σ = 0.71 × 10^−5^ S/cm for the as-prepared film to σ = 5.25 × 10^−5^ S/cm for the films annealed at 400 °C. It was observed that the transmittance of the 300 °C annealed film was similar to that of the substrate (approximately 85%), which may be the result of less light scattering loss due to the smoother and denser film surface [78].

Abhilash [76] obtained Li_0.5_La_0.5_TiO_3_ thin films by spin coating. The films were calcinated at 550 °C for 40 h to obtain the LLTO tetragonal phase. The space group in the phase crystallized is the ubiquitous P4/mmm and the lattice parameters are a = 3.887 Å and c = 7.764 Å (c ≈ 2a). The size of the crystallites was 25 nm for the sample calcinated for 20 h and, respectively, 34 nm for the sample calcinated for 40 h. The ionic conductivity of the thin films increases slightly after calcination from σ = 3.10 × 10^−7^ S/cm at 550 °C for 5 h up to σ = 3.52 × 10^−7^ S/cm for calcination time of 40 h [76].

Aguesse [77] prepared Li_3x_La_2/3−x_TiO_3_ thin films by PLD on STO, LAO and MgO substrates. The authors highlighted the importance of the deposition parameters (namely the partial oxygen pressure) on the phase purity of the resulting thin films. TiO_2_ is found as a secondary product. Thus, it was observed that LLTO films without the secondary TiO_2_ phase can only be formed on a narrow range of oxygen pressure, between 10 and 15 Pa [77]. The substrate temperature is another critical parameter. The authors observed that at lower deposition temperatures the films present a large microstrain, suggesting the existence of defects or composition segregation due to local stress. High deposition temperatures are, therefore, favoured for high crystallinity of the LLTO thin film. The films present the tetragonal crystallographic structure with the space group P4/mmm and the lattice parameters a = 3.8741 Å and c = 7.7459 Å. The films were prepared from targets with the Li_0.37_La_0.54_TiO_3_ composition to which different amounts of excess Li were added. The conductivity of the films prepared from targets containing 10 and 20 mol% excess Li is higher than the conductivity obtained for films prepared from targets containing 5 mol% excess Li [77].

Lee [79] prepared LLTO solid electrolyte thin films with thicknesses between 100 and 200 nm by radio frequency magnetron sputtering. Here LLTO has the composition Li_5_La_3_Ta_2_O_12_. The thin film electrolyte has an ionic conductivity of 7.6 × 10^−6^ S/cm.

The effects of the Sr substitution were also tested on thin films by Shui [7]. The thin films were obtained by magnetron sputtering from a ceramic target with the Li_0.43_La_0.457_Sr_0.1_TiO_3_ target [7]. Some unknown phase was observed in the film XRD patterns. Otherwise, the shift in the peak positions indicate that Sr enters the LLTO structure [7] in agreement with the literature results [125,126]. It was observed that film resistivity increases with the film annealing temperature. At the same time the ionic conductivity of the thin films is improved with the increase in the annealing temperature [7] and reaches a maximum of 4.63 × 10^−5^ S/cm at 300 °C. Further increase in annealing temperature (at 400 °C) lowers ionic conductivity, possibly due to some reaction with the substrate [7]. On the other hand, the ionic conductivity of the Sr doped LLTO thin films is higher than the ionic conductivity of pristine LLTO films and LLTO films doped with Al or Ge [7].

## 8. Conclusions

The Li_3x_La_2/3−x_TiO_3_ compounds present ionic conductivity properties defined by a set of strongly correlated parameters. These parameters range from microscopic characteristics (such as grain boundaries) to atomic scale properties (such as ionic radii, oxidation states). On the other hand, the experimentally available synthesis parameters, which are used to indirectly tune the ionic conductivity of the material are rather few (chemical composition, sintering time and sintering temperature). Due to the interdependency of these parameters, optimization of ionic conductivity is not a linear process, meaning that adjustment of an experimental parameter for improving one component of the material’s ionic conductivity is also, at the same time, worsening another component. On a top–down approach, the material parameters which dictate the ionic conductivity are: chemical composition, concentration of charge carriers, concentration of vacancies, number and type of grain boundaries (crystallite size, shape, degree of packing), porosity, number and type of domain boundaries, type of crystal symmetry, unit cell volume, crystal structure, ionic radii and type of chemical bonds.

For example, increasing sintering temperature/sintering time favours the crystallite growth which reduces grain boundaries and improves conductivity. At the same time, Li evaporation increases, leading to loss of charge carriers and increased porosity, which lower conductivity.

High cooling speed leads to the formation of higher symmetry (cubic) crystal systems which have higher conductivity, but limits the grain size growth, which increases the number of grain boundaries and hence lowers conductivity. Slow cooling results in large grains, but lower symmetry (tetragonal or orthorhombic) crystallization and formation of superstructure stacking which causes anisotropic, lower conductivity.

The material presents crystallographic domains rotated by 90°. The resistivity to ion conduction at the domain boundaries is very high. It also seems to be inversely proportional to the domain boundary concentration. DB resistivity and concentration compete with each other. Conductivity is higher for smaller domains because the smaller domains present lower resistivity. The size of the domains decreases when the Li concentration increases and when the sintering temperature is lower, hence the ionic conductivity improves for high Li content and low sintering temperature. However, the lower sintering temperature limits the grain size, and this increases grain boundary concentration which again limits conductivity.

At the atomic scale, the conductivity within the crystal is dependent on many parameters such as the unit cell size, the dimensions and tilt of the TiO_6_ octahedra, which is again determined by the type of chemical bond between the B-site ion (Ti^4+^ and the Ti^4+^ substitution cation) and the anion (O^2−^ and O^2−^ substitution anion, typically F^−^). Substituting the La^3+^ ion with a higher ionic radius element increases the unit cell volume and the space available for Li transport. However, at the same time it also lowers the concentration of vacancies which again, lowers conductivity.

An overview of the correlation between some material parameters is tentatively represented in Table 7.

Various optimization strategies result in markedly different total ionic conductivity values. Some of the best results are reviewed in Table 8.

Disadvantages of the LLTO material for usage as solid state electrolyte include reduction of Ti^4+^ to Ti^3+^ when the electrolyte is brought in contact with metallic Li and possibly during Li transport. The reduction leads to increased electronic conductivity. The material’s electronic conductivity is seen to increase with the temperature, which limits the operating temperature of potential batteries fabricated with an LLTO solid state electrolyte. Moreover, the material is susceptible to degradation through Li^+^/H^+^ exchange if it is stored in neutral or acidic environments.

Then, the LLTO compounds could find other applications, in addition to Li battery electrolytes and electrodes. For example, the ability of the material to exchange Li^+^ ions with H^+^ ions can be exploited in the design of new sensors.

The study of the material could also be interesting even from the perspective of fundamental research. Some information can be inferred on ionic radii for Na^+^ and, respectively, Ag^+^ in the LLTO matrix. From the results of the Li^+^ substitution with the two cations, assuming all else is equal, it seems that the Ag^+^-ion radius is smaller than the Na^+^ ion (since Ag^+^ can be transported through the LLTO lattice, while Na^+^ is fixed).

From the perspective of the main application (lithium batteries), both SSEs and CPEs based on LLTO enable the fabrication of batteries with performances comparable to the ones achieved by conventional batteries. Table 9 indicates some of the battery parameters measured for different classes of devices.

Future research directions in the field of LLTO could include identifying additional synthesis methods, which could enable greater yield at lower cost, mapping the interdependence of the material properties and their corelated effects on the ionic conductivity. It is also possible to further investigate the effects of substitutions on both A- and B-sites of the perovskite structure. Such endeavours could generate vast amounts of information. As the information technology now allows unprecedented computing power, artificial intelligence and simulations will most likely contribute to reducing the time required for testing every possible combination and finding the optimum composition for the material. Such approaches are already implemented [128].

## Figures and Tables

**Table 1:** Properties of main type of electrolytes.

Property	Polymer	Inorganic (Ceramic)	Hybrid/Composite
ionic conductivity	good at high temperature (60 °C)	high even at RT	high
mechanical strength	poor	excellent	medium
interface	good (low impedance)	poor (high impedance)	good

**Table 3:** Typical reaction conditions for the synthesis of LLTO by the sol-gel method.

Final Product	Reagents	Gel Formation	Calcination	Sintering	Ref.
pristine LLTO and Sr doped LLTO Li_0.35_La_0.55_TiO_3_ Li_0.35_La_0.35_Sr_0.03_TiO_3_	Sr(NO_3_)_2_, La_2_O_3_, LiNO_3_, C_6_H_8_O_7_×H_2_O	80 °C for 2 h combustion at 250 °C	650 °C for 6 h	1250 °C for 4 h	[6]
Li_0.35_La_0.55_TiO_3_	Ti, HCl, C_4_H_6_O_6_ (tartaric acid), LiNO_3_, La_2_O_3_	90 to 120 °C	800, 900, 1000 and 1100 °C	1250 °C	[51]
Eu doped Li_0.5_La_0.5_TiO_3_	Ti[OCH(CH_3_)_2_]_4_, LiNO_3_, La(NO_3_)_3_×6H_2_O, Eu_2_O_3_, C_6_H_8_O_7_ (citric acid), (CH_2_OH)_2_ (ethylene glycol)	120 °C for one hour	350 °C for 2 h	800 °C for 3 h	[52]
Al doped LLTO (Li_0.33_La_0.56_)_1.005_Ti_0.99_Al_0.01_O_3_	LiNO_3_, La_2_(NO_3_)_3_×6H_2_O, Ti[OCH(CH_3_)_2_]_4_, Al(NO_3_)_3_×9H_2_O, (CH_2_)_2_(OH)_2_, C_6_H_8_O_7_, Li_2_O	70 °C for 12 h to form a gel then heating to 100 °C to form a resin	350 °C for 6 h + 750 °C for 3 h	1350 °C for 6 h	[53]
Li_0.35_La_0.55_TiO_3_	LiNO_3_, La(NO_3_)_3_×6H_2_O, Ti(OC_4_H_9_)_4_	80 °C for gel formation and drying at 150 °C	combustion at 350 °C for 4 h + calcination at 900 °C for 2 h	no sintering	[54]
Li_0.33_La_0.56_TiO_3_	La(NO_3_)_3_×4H_2_O, LiNO_3_, Ti(OC_3_H_7_)_4_	95 °C for 2 h and 100 °C for 12 h	combustion at 450 °C for 30 min + calcination at 800–1200 °C for 12 h	1150 °C for 10 h	[32]

**Table 4:** Typical reaction conditions for the synthesis of LLTO by solid state reaction.

Final Product	Reagents	Calcination	Sintering	Ref.
Li_0.34_La_0.51_TiO_2.94_	La_2_O_3_, Li_2_CO_3_, TiO_2_	800 °C for 4 h + two heat treatments with intermediary grinding at 1150 °C for 12 h	1350 °C for 6 h	[61]
Li_0.33_La_0.56_TiO_3_	Li_2_CO_3_, La_2_O_3_, TiO_2_	800 °C for 8 h	1250 °C + 1350 °C for 12 h followed by quenching	[57]
Li_0.33_La_0.56−y_TiO_3−3y_F_3y_ (y = 0.017, 0.05) Li_0.33+3y_La_0.56−y_TiO_3_ (y = 0, 0.01, 0.02, 0.04)	Li_2_CO_3_, La_2_O_3_, TiO_2_, LiF	650 °C for 12 h	1350 °C for 1.5 h followed by quenching	[5]
Li_0.5−x_La_0.5_Na_x_TiO_3_	Li_2_CO_3_, Na_2_CO_3_, La_2_O_3_, TiO_2_	1100 °C for 4 h	1300–1330 °C for 6 h	[69]
LLTO doped with rare earths	La_2_O_3_, Li_2_CO_3_, Na_2_CO_3_, TiO_2_, SrCO_3_, BaCO_3_, MgO	800 °C for 4 h, 1150–1200 °C for 6 to 12 h with intermediary grinding	1350–1400 °C for 3 to 10 h	[73]
Li_0.33_La_0.56_TiO_3−y_F_y_ 0 ≤ y ≤ 0.183	La_2_O_3_, TiO_2_, Li_2_CO_3_, LiF	800 °C for 2 h	1200 °C for 10 h	[74]

**Table 5:** Thin film deposition parameters.

Method	Parameters	Observations	Ref.
e-beam evaporation	LLTO target chamber pressure 7 × 10^−2^ Pa O_2_/Ar ratio 1:2 target to substrate distance 30 cm beam power 300–600 W		[67]
PLD KrF laser 248 nm wavelength, aimed at 45° on rotating target	Li_x_La_2/3−x_TiO_3_ (x = 0.1, 0.2, 0.3, 0.4, 0.5) target chamber pressure: 1 × 10^−6^ to 5 × 10^−6^ torr target substrate distance 59 mm substrate temperature: RT pulse frequency: 10 Hz laser power: 180 mJ/pulse	amorphous film	[64]
RF magnetron sputtering	chamber pressure: 1 Pa O_2_/Ar ratio 30% O_2_, 70% Ar magnetron power 80 W		[78]

**Table 6:** Review of the possible crystallization systems that LLTO can take.

Composition	Synthesis Conditions	Crystallization	Lattice Constants	Ref.
Li_0.35_La_0.567_TiO_3_	800 °C	Tetragonal P4/mmm	a = 5.48869 c = 7.71678	[51]
Li_0.35_La_0.567_TiO_3_	900 °C		a = 5.47065 c = 7.77080	
Li_0.35_La_0.567_TiO_3_	1000 °C		a = 5.47239 c = 7.77586	
Li_0.35_La_0.567_TiO_3_	1100 °C		a = 5.49422 c = 7.75065	
Li_0.33_La_0.556_TiO_3_	1250 °C			[62]
Li_0.4_La_0.533_TiO_3_	1250, 1300 °C			
Li_0.26_La_0.61_TiO_3_	1000 °C		a = 3.875 c = 7.739	[56]
Li_0.33_La_0.556_TiO_3_	Slow cooling			[57]
Li_0.438_La_0.52_TiO_3_			a = 3.87 c ≈ 2a	
Li_0.501_La_0.499_TiO_3_			a = 3.87 c ≈ 2a	
Li_0.5_La_0.5_TiO_3_	1350 °C		a = 3.6 c = 7.2	[87]
Li_0.5_La_0.5_TiO_3_	900 °C		a = 3.93 c = 7.86	[88]
Li_0.35_La_0.55_TiO_3_	1100 °C	Orthorhombic Pmmm Cmmm	a = 7.74351 b = 7.74209 c = 7.73723	[51]
Li_0.35_La_0.55_TiO_3_	1250 °C		a = 7.74610 b = 7.73459 c = 7.73991	
Li_0.16_La_0.613_TiO_3_	1250, 1300, 1350 °C	Orthorhombic Pmmm		[62]
Li_0.33_La_0.556_TiO_3_	1300, 1350 °C			
Li_0.4_La_0.533_TiO_3_	1350 °C			
Li_0.09_La_0.636_TiO_3_			a = 3.864 b = 3.875 c = 7.786	[57]
Li_0.189_La_0.603_TiO_3_				
Li_0.29_La_0.57_TiO_3_	1400, 1460 °C	Orthorhombic Cmmm	a = 7.737(1) b = 7.742(1) c = 7.785(1)	[63]
Li_0.33_La_0.556_TiO_3_	quenching	Cubic Pm3m		[57]

**Table 7:** Interdependence of material properties.

Main Material Characteristic	Effect on Conductivity	Variables Determining the Main Material Parameter	Other Parameters Affected by Optimization of Main Material Characteristic	Requirement for Improving Conductivity
grain boundary	decrease	sintering temperature, cooling speed	Li^+^ concentration, crystal symmetry	high temperature, slow cooling
domain boundary	decrease	sintering temperature, Li content	Li^+^ concentration, grain boundary	low sintering temperature, high Li content
crystal symmetry	increase if symmetry is high	sintering temperature, cooling speed, Li content	Li^+^ concentration, grain boundary	high temperature, quenching
crystal structure	increase if more space is available for Li transport	chemical composition, synthesis parameters	unit cell volume, bottleneck size, TiO_6_ octahedron size and tilt, Li^+^ concentration, vacancy concentration	increase La^3+^ substitution degree (with larger radius cation)
Li^+^/vacancy concentration	linear	chemical composition, synthesis parameters, reaction dynamics	crystal structure, grain boundary	decrease La^3+^ substitution degree (with larger radius cation)

**Table 8:** Overview of ionic conductivity values.

Sample	Highest Conductivity at RT (S/cm)	Ref.
liquid organic electrolyte	~10^−2^	[6]
large grain size	4.86 × 10^−4^	[57]
pristine Li_0.35_La_0.55_TiO_3_	9.15 × 10^−4^	[6]
Sr^2+^/La^3+^ substitution	1.5 × 10^−3^ up to 1.95 × 10^−3^	[6,73]
Ti^4+^/Al^3+^ substitution	3.17 × 10^−4^	[1]
O^2−^/F^−^ substitution	2.3 × 10^−3^ (30 °C)	[5]
PVDF/LLTO (15% LLTO wt.)	5.8 × 10^−4^	[107]
PEO/LLTO (5% LLTO wt.)	5.53 × 10^−5^ (25 °C) and 4.65 × 10^−4^ (60 °C)	[108]
Vr-LLTO/PEO	1.04 × 10^−4^ (25 °C)	[119]
PE-LLTO	0.38 × 10^−3^	[123]
LLTO-PEO heterostructure	1.49 × 10^−4^ (30 °C)	[120]

**Table 9:** Battery performance.

Battery/Electrolyte	Initial Specific Capacity	Capacity Retention	Ref.
PVDF-LLTO	≈150–170 mAh/g	140 mAh after 200 cycles	[107]
LLTO coating for cathode material	135 mAh/g	80% after 200 cycles	[118]
PEO/LiTFSI/LLTO	≈135 mAh/g	123 mAh/g after 100 cycles at 60 °C	[108]
LLTO-PE	≈150.3 mAh/g	88.7% after 1000 cycles	[123]
Li|LLTO-PVDF|LiFePO_4_	>150 mAh/g	91.7% after 1000 cycles	[2]
Metallic Li battery|fluorinated LLTO-PEO electrolyte	>125 mAh/g	>80% after 100 cycles at 90 °C	[122]
Al-LLTO aqueous battery Li metal anode, LiCoO_2_ cathode	150–200 mAh/g	59.3% after 100 cycles	[1]
Organic type Li-oxygen battery, Al-LLTO electrolyte	300 mAh/g	100% after 100 cycles	[53]
LiTFSI-BL-SN	170–180 mAh/g	90% after 500 cycles	[24]
SiO_x_/C anode	800–830 mAh/g	600–700 mAh/g after 40–50 cycles at a rate of 2 C 800 mAh after 100 cycles at a rate of 0.1 C	[25]
Metallic Li battery, Li_4_Ti_5_O_12_ electrode/PVDF electrolyte	150 mAh/g	119 mAh/g after 400 cycles at a rate of 5 C	[27]
Typical phone battery	372 mAh/g		[127]

## Data Availability

Data sharing not applicable. No new data were created or analysed in this study.
